# Supplementary material for: Genome Sequencing Reveals Loci under Artificial Selection that Underlie Disease Phenotypes in the Laboratory Rat
Source: Cell. 2013 Aug 1;154(3):691–703. doi: 10.1016/j.cell.2013.06.040 (PMC3732391; doi:10.1016/j.cell.2013.06.040)
Supplement: Document S1. Article plus Supplemental Information [file mmc10.pdf]

Resource

Cell

Genome Sequencing Reveals Loci under Artificial Selection that Underlie Disease Phenotypes in the Laboratory Rat

Santosh S. Atanur,[1,2] Ana Garcia Diaz,[1] Klio Maratou,[1] Allison Sarkis,[5] Maxime Rotival,[3] Laurence Game,[4] Michael R. Tschannen,[5] Pamela J. Kaisaki,[6] Georg W. Otto,[6] Man Chun John Ma,[7] Thomas M. Keane,[8] Oliver Hummel,[9] Kathrin Saar,[9] Wei Chen,[9] Victor Guryev,[10,11] Kathirvel Gopalakrishnan,[12] Michael R. Garrett,[13] Bina Joe,[12] Lorena Citterio,[14] Giuseppe Bianchi,[14] Martin McBride,[15] Anna Dominiczak,[15] David J. Adams,[8] Tadao Serikawa,[16] Paul Flicek,[17] Edwin Cuppen,[10] Norbert Hubner,[9,18] Enrico Petretto,[3] Dominique Gauguier,[6,19] Anne Kwitek,[7] Howard Jacob,[5] and Timothy J. Aitman[1,*]
[1]Physiological Genomic and Medicine Group, MRC Clinical Sciences Centre
[2]National Heart and Lung Institute
[3]Integrative Genomics and Medicine Group, MRC Clinical Sciences Centre
[4]Genomics Core Laboratory, MRC Clinical Sciences Centre
Imperial College London, London W12 0NN, UK
[5]Department of Physiology, Medical College of Wisconsin, Milwaukee, WI 53226, USA
[6]The Welcome Trust Centre for Human Genetics, University of Oxford, Oxford OX3 7BN, UK
[7]Department of Pharmacology, University of Iowa, Iowa City, IA 52242, USA
[8]The Wellcome Trust Sanger Institute, Hinxton, Cambridge CB10 1SA, UK
[9]Max Delbruck Center for Molecular Medicine, Berlin 13092, Germany
[10]Hubrecht Institute KNAW and University Medical Center Utrecht, Uppsalalaan 8, 3584 Utrecht, the Netherlands
[11]European Research Institute for the Biology of Ageing, University Medical Center, 9700 AD Groningen, the Netherlands
[12]Center for Hypertension and Personalized Medicine, Department of Physiology and Pharmacology, University of Toledo College of Medicine, Toledo, OH 43606-3390, USA
[13]Department of Pharmacology and Toxicology, University of Mississippi Medical Center, Jackson, MS 39216, USA
[14]San Raffaele Scientific Institute, OU Nephrology, University Vita Salute San Raffaele, Chair of Nephrology, 58, 20132 Milan, Italy
[15]Institute of Cardiovascular and Medical Sciences, University of Glasgow, Glasgow G12 8QQ, UK
[16]Institute of Laboratory Animals, Graduate School of Medicine, Kyoto University, Kyoto 606-8501, Japan
[17]European Molecular Biology Laboratory, European Bioinformatics Institute, Wellcome Trust Genome Campus, Hinxton, Cambridge CB10 1SD, UK
[18]DZHK (German Centre for Cardiovascular Research), Partner Site Berlin, Berlin 13092, Germany
[19]INSERM UMR-S872, Cordeliers Research Centre, 75006 Paris, France
*Correspondence: t.aitman@csc.mrc.ac.uk
http://dx.doi.org/10.1016/j.cell.2013.06.040
This is an open-access article distributed under the terms of the Creative Commons Attribution License, which permits unrestricted use, distribution, and reproduction in any medium, provided the original author and source are credited.

SUMMARY

Large numbers of inbred laboratory rat strains have been developed for a range of complex disease phenotypes. To gain insights into the evolutionary pressures underlying selection for these phenotypes, we sequenced the genomes of 27 rat strains, including 11 models of hypertension, diabetes, and insulin resistance, along with their respective control strains. Altogether, we identified more than 13 million single-nucleotide variants, indels, and structural variants across these rat strains. Analysis of strain-specific selective sweeps and gene clusters implicated genes and pathways involved in cation transport, angiotensin production, and regulators of oxidative stress in the development of cardiovascular disease phenotypes in rats. Many of the rat loci that we identified overlap with previously mapped loci for related traits in humans, indicating the presence of shared pathways underlying these phenotypes in rats and humans. These data represent a step change in resources available for evolutionary analysis of complex traits in disease models.

INTRODUCTION

In the past 100 years, more than 500 inbred rat strains have been derived for a range of physiological and pathophysiological phenotypes (Aitman et al., 2008; Lindsey, 1979) but have been predominantly used to study cardiovascular and metabolic phenotypes, which are complex traits governed by the interaction between multiple genetic factors and the environment. Inbred rat models of cardiovascular and metabolic phenotypes have been derived from various founder colonies or stocks at

various geographic locations by crossing relatively small numbers of rats within the colony and selecting for the desired disease phenotypes over several generations, with simultaneous or subsequent brother-sister mating to develop genetically homogeneous inbred strains (Jong, 1984; Rapp, 2000).

Although the majority of inbred rat models of hypertension and diabetes were generated from outbred Wistar colonies, efforts have been made to derive disease models on various other genetic backgrounds (Jong, 1984). Each strain so derived is therefore expected to show major founder effects and should be genetically and phenotypically distinct from its founder colony as well as from other strains derived from different founder colonies (Doggrell and Brown, 1998). Significant genotypic and phenotypic heterogeneity in the genetic models of hypertension and diabetes therefore provides a unique resource to study molecular mechanisms behind different etiological forms of hypertension. In addition, metabolic phenotypes such as insulin resistance and dyslipidaemia, which were frequently co-inherited with hypertension and may form part of the hypertension phenotype, may have inadvertently been coselected with hypertension, as well as compensatory alleles that protect against target organ damage mediated by phenotypes such as hypertension (St Lezin et al., 1999).

We hypothesize that, in these rat strains, phenotype-driven selection may have resulted in "artificial selective sweeps" with fixation of sequence variants that underlie disease phenotypes, as has been observed in the artificial selection of a number of other disparate but benign traits in different species (Rubin et al., 2010; Wright et al., 2005; Xia et al., 2009). Artificial selective sweeps in inbred rat strains may be unique to a particular strain and disease phenotype in comparison with other strains and may contribute to the molecular basis of hypertension and other related phenotypes in that strain.

Genes in a genome evolve at different evolutionary rates due to varying evolutionary constraints on each gene, and interactions between genes underlying complex phenotypic traits are maintained by coevolution (Pagliarini et al., 2008; Tillier and Charlebois, 2009). Because of the polygenic nature of hypertension and other complex phenotypes, genes containing disease-inducing variants might have evolved together at an evolutionary rate that will be different from the rest of the genome. The identification of genes that have coevolved and of artificial selective sweeps in rat disease models may therefore be informative for identifying loci underlying disease phenotypes and for understanding the polygenic architecture of complex disease phenotypes.

Present sequencing technology now permits rapid and accurate sequencing of whole genomes through which near-complete catalogs of genomic variants can be obtained. It is therefore possible to perform, in model organisms, genomic screening for identification of coevolved gene clusters and artificial selective sweeps that harbor potentially pathogenic genes and mutations.

In this study, we sequenced the genomes of 25 new rat strains on high-throughput sequencing platforms, with a major focus on strains with well-characterized cardiovascular and metabolic phenotypes. These included 11 widely used rat models of hypertension, diabetes, and insulin resistance, along with their respective control strains. We identified a comprehensive catalog of genomic variants in 27 rat strains (25 strains sequenced for this study and two strains sequenced previously; Atanur et al., 2010; Simonis et al., 2012). We also identified clusters of genes and genomic loci that coevolved during selective breeding of these laboratory rat strains and established their relationship to genes and loci known to underlie disease phenotypes in these strains. The study therefore provides insights into the genetics and biology of cardiovascular and metabolic phenotypes shown by the sequenced rat strains.

RESULTS

Sequencing and Variant Calling

We sequenced the genomes of 25 rat strains and analyzed them together with the genomes of two rat strains that we sequenced previously (Atanur et al., 2010; Simonis et al., 2012). We also resequenced the genome of BN/Mcwi to correct for errors in the reference genome, using DNA from the same animal that was used for the reference BN genome sequence (Gibbs et al., 2004). Quality filtered sequence reads were mapped to the reference BN/Mcwi genome assembly (referred to hereafter as the BN reference), version RGSC3.4. After removing clonal reads, we achieved at least or close to 20× coverage for all strains except for strains BBDP/Wor and WKY/NHsd, for which we achieved ∼10× coverage (Table 1).

After applying rigorous filtering criteria (see Extended Experimental Procedures available online) and excluding genomic variants that arose due to potential errors in the reference genome (in which the reference allele was different from the BN/Mcwi allele detected by resequencing), 9,665,340 single-nucleotide variants (SNVs) and 3,502,117 short indels were identified across the 27 rat strains (Table 1). This includes 839,691 SNVs and 479,974 indels, in which we could not determine the BN/Mcwi allele by resequencing because of either low coverage or poor read mapping and/or base quality. The variant set therefore may still contain a small proportion (0.001%) of false positives due to base call errors in the reference BN genome. We found that 98.3% of SNV calls were homozygous and 1.7% were heterozygous, the latter most likely arising from a combination of true heterozygote loci due to incomplete fixation of the inbred strain and false-positive calls due to regions of copy number variation, sequencing errors, and mapping errors in highly repetitive genomic regions, as previously described (Atanur et al., 2010; Keane et al., 2011). Along with SNVs and indels, we also identified a total of 719,929 structural variants in the 26 rat strains sequenced on the Illumina platform.

To assess the sensitivity and specificity of the variant calls, we compared the LE/Stm Illumina sequence with the publicly available sequence of 13 bacterial artificial chromosomes (BACs) derived from the LE/Stm genome generated by conventional capillary sequencing, which provided a control sequence region of ∼2 Mb for LE/Stm. There were 3,382 SNVs and 1,211 indels identified in the LE/Stm BAC sequences compared to the BN reference genome. We estimated 0.38% false-positive and 8.1% false-negative SNV calls in the Illumina LE/Stm sequence, with an estimated false positive and false negative rate for indels of 3.75% and 10.98%, respectively. Thus the overall accuracy of genotype calls, including variant and reference calls, was

Table 1. Sequencing Details and Variant Calls in 27 Inbred Rat Strains

Rat Strain	Gb of Bases Mapped[a]	Average Coverage[a]	Number of SNVs[b]	Number of Indels[b]	Number of Structural Variants[b]
ACI/EurMcwi	86.64	33.69	3,607,275	1,168,780	17,859
BBDP/Wor	25.68	9.99	3,322,410	1,131,697	35,387
BN.Lx	58.40	22.71	51,938	45,404	NA
F344/NCrl	65.13	25.33	3,433,241	1,132,993	42,138
FHH/EurMcwi	57.98	22.55	3,471,696	1,170,337	22,628
FHL/EurMcwi	53.25	20.71	3,422,550	1,117,039	14,217
GK/Ox	75.25	29.26	3,584,504	1,147,996	58,877
LE/Stm	56.11	21.82	3,485,480	1,152,163	19,285
LEW/NCrlBR	51.06	19.86	2,966,945	1,014,796	39,031
LEW/Crl	66.30	25.78	2,941,368	1,002,364	39,146
LH/MavRrrc	55.84	21.71	3,459,239	1,189,791	21,901
LL/MavRrrc	58.04	22.57	3,419,697	1,177,989	20,623
LN/MavRrrc	56.54	21.99	3,406,103	1,171,795	20,561
MHS/Gib	64.68	25.15	3,270,047	1,112,526	23,551
MNS/Gib	57.26	22.27	3,278,667	1,123,980	22,769
SBH/Ygl	65.76	25.57	3,461,088	1,134,320	13,789
SBN/Ygl	47.17	18.34	3,334,857	1,078,851	9,586
SHR/NHsd	61.25	23.82	3,795,348	1,222,619	45,284
SHR/OlaIpcv	52.72	20.5	3,832,318	1,276,189	11,781
SHRSP/Gla	70.03	27.23	3,735,521	1,174,933	14,665
SR/Jr	50.28	19.55	3,421,364	1,140,725	36,798
SS/JrHsdMcwi	55.82	21.71	3,383,380	1,136,032	11,124
SS/Jr	51.42	19.99	3,377,708	1,110,653	38,535
WAG/Rij	53.58	20.84	3,167,781	1,084,318	51,423
WKY/Gla	68.17	26.51	3,819,860	1,234,622	11,310
WKY/NHsd	30.02	11.67	3,877,157	1,313,104	31,875
WKY/NCrl	63.77	24.8	3,718,449	1,250,041	45,788

See also Figure S1 and Tables S1 and S2.

[a]Gb of bases mapped and average coverage were calculated after removing clonal reads.

[b]Numbers of sequence variants relative to the BN reference genome.

99.99% for the LE/Stm genome, which can be scaled to the remaining 25 rat strains sequenced on the Illumina platform, as the variants were called in these strains simultaneously with the same methods.

Within the total set of 9,665,340 identified SNVs, 67,616 were in protein-coding sequence, among which 29,131 were nonsynonymous coding (NSC) variants and 38,485 were synonymous coding (SC) variants. Of the 29,131 NSC variants, 409 predicted premature truncation of the protein due to gain of stop codons, whereas 27 predicted the loss of stop codons (Table S1 and Figures S1A and S1B). We also identified 2,366 short indels in protein-coding sequence, which predicted disruption of the open reading frame of the encoded protein (Table S2 and Figures S1C and S1D). Of the observed 37,510 large deletions, 1,497 overlapped with the predicted protein-coding sequence.

Phylogenetic History of Laboratory Rat Strains

The evolutionary history of laboratory rat strains is complex, as they were derived by multiple rounds of interbreeding and inbreeding in different locations at different points in time (Jong, 1984; Saar et al., 2008). To inform understanding of the evolutionary history of laboratory rat strains, we constructed a phylogenetic tree of strains, including the BN reference strain, using our catalog of 9.6 million high-quality SNVs. Laboratory rat strains clustered essentially according to founder stocks and colonies from which they were derived (Figure 1). Moreover, Wistar-derived strains showed two prominent subclusters, consistent with the known breeding history of Wistar-derived strains in Japan (SHR, SHRSP, GK, and WKY) or elsewhere in Europe and the USA (MHS, MNS, LEW, WAG, and BBDP). Sprague-Dawley-derived (SD) strains (SS, SR, LH, LL, and LN) also clustered together, whereas other strains formed more isolated clusters, including BN strains from which the reference genome strain (BN/Mcwi) was selected. The WKY/Gla strain was closer to SHR substrains than other WKY substrains, possibly reflecting the known diversity of WKY strains arising from their distribution to different geographical locations before complete inbreeding (Kurtz et al., 1989) or from the close relationship of SHR and WKY sublines during their establishment and maintenance.

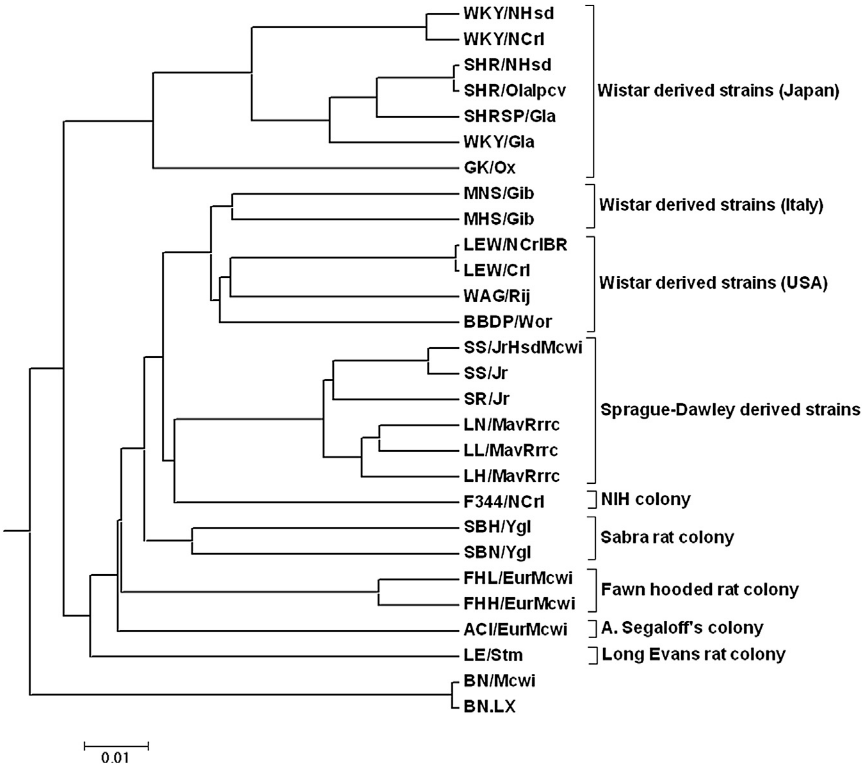

Figure 1. Phylogenetic Tree of 28 Rat Strains

The phylogenetic tree was constructed using 9.6 million SNVs across 28 laboratory rat strains, including the Brown Norway reference strain (BN/Mcwi). The scale represents genetic distance; the distance matrix was calculated by dividing the number of SNVs between a given pair of strains by the length of the BN reference genome. The phylogenetic tree was constructed using the Fitch-Margoliash method with 1,000 bootstraps.

Coevolutionary Gene Clusters

Because rat models of hypertension, insulin resistance, and diabetes were selectively bred for these genetically complex disease phenotypes, the genes underlying these phenotypes may have been coselected and coevolved because of the selective pressure exerted on them during the derivation of these strains. Functionally related genes with a similar evolutionary rate tend to have a similar phylogenetic history (Pagliarini et al., 2008). To identify clusters of genes that coevolved during selection for complex traits, we used a mirror tree approach (Pazos and Valencia, 2001), as illustrated in Figure 2, to test the hypothesis that genetic effects on disease phenotypes may be detectable as coevolved variation in protein-coding sequence between rat strains. Coevolutionary gene clusters were identified using transcripts that show NSC variants, including SNVs causing a stop gain or stop loss in at least one strain compared to the reference BN genome. We calculated evolutionary rate by dividing the number of NSC variants in a transcript by the number of SC variants in the same transcript, both normalized by the length of the transcript. This defined a phylogenetic vector for each transcript. Similarly phylogenetic vectors were calculated for each transcript showing frameshift due to indels.

To take into account the effect of population structure on evolutionary rate, we used SNV data across the strains to estimate principal components contributing to variability in the strains (Sato et al., 2005). The first two principal components were found to explain ~70% of the sequence variability across strains (Figure S2A) and clearly separate Japanese Wistar-derived strains, SD-derived strains, and the remaining rat strains (Figure S2B). We adjusted the evolutionary rate for the first two principal components and then calculated correlation coefficients between the residuals for all possible pairwise combinations (89,024,496) of transcripts containing NSC SNVs. Coevolutionary clusters were then identified using 310,690 pairs of transcripts with significant correlation ($|r| > 0.96$; p value $< 9.33 \times 10^{-15}$; FDR < 0.1%). A total of 1,955 clusters were identified (Table S3A), the majority of them containing two (n = 1063) or three (n = 383) transcripts, whereas 109 clusters contain 10 or more transcripts (Table S3A). Confirming the cluster analysis, for 1,133 clusters, more than 40% of the NSC mutations represented within the cluster showed linkage disequilibrium (LD; $r^2 > 0.8$) with each other, and the majority of them showed perfect LD ($r^2 = 1$) even though SNVs were located on different chromosomes. After random shuffling of the SNVs, this LD structure was completely lost (Figure S3), suggesting that mutations in these genes may have coevolved under the same selection pressures. Relatively smaller numbers (n = 145) of clusters were identified using frameshift coding mutations (Table S3B) because only 1,634 of the transcripts show frameshift coding mutations in at least one strain compared to the BN reference genome.

To corroborate the data generated from principal component analysis (PCA), we also used the R package EMMA (Kang et al., 2008), which is based on a linear mixed model to correct for population structure and genetic relatedness in model organism association mapping, to identify coevolutionary gene clusters. The networks obtained from EMMA showed high concordance with the networks obtained from PCA-based population structure correction, with ~75% of edges shared between the two networks (p value for significant overlap $< 10^{-16}$). The number of shared edges was raised to an average of 94% (min 72%, max 100%) when considering edges in clusters identified from PCA-derived networks that were unique to a single strain (Table S4).

The majority of the large clusters contain transcripts mutated either uniquely in a single strain or in strains that were derived from the same founder colony and show close genetic proximity in the phylogenetic tree. For example, the two fawn-hooded rat strains FHH and FHL shared NSC variants in 311 transcripts that were unique just to these strains, and the Wistar-derived

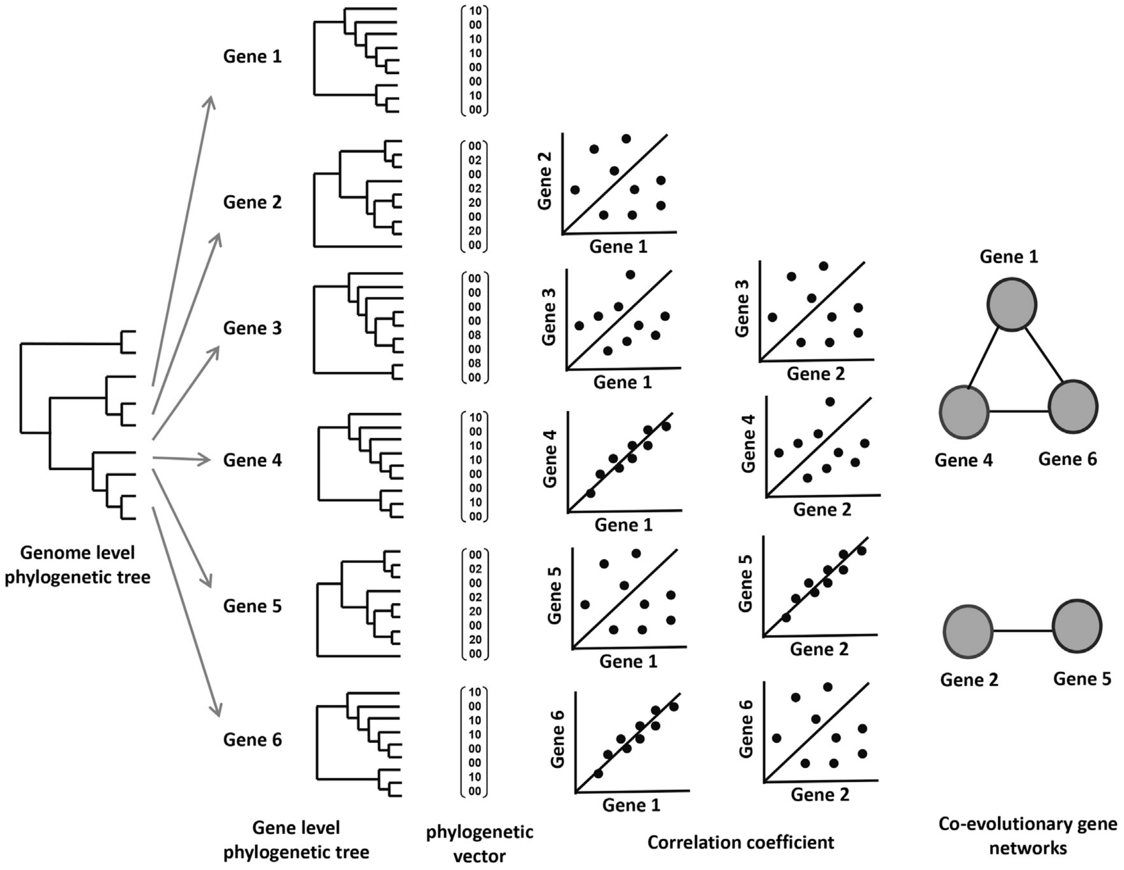

Figure 2. Illustration of the Mirror Tree Approach

In this illustration, hypothetical genes 1, 4, and 6 evolved together, as they show identical phylogenetic history, whereas genes 2 and 5 also show an identical evolutionary history, though these two groups of genes evolved at a different evolutionary rate. These evolutionary patterns of individual genes were converted into phylogenetic vectors. Phylogenetic vectors of coevolving genes such as genes 1, 4, and 6 are more highly correlated than those that evolved at different rates such as genes 2 and 6. Networks of genes that coevolved were generated from significantly correlated genes. Phylogenetic vectors were derived by taking into account population structure.

See also Figure S2 and Tables S3, S4, and S8.

SHR, SHRSP, and WKY strains uniquely shared NSCs in 140 transcripts (Table S3A). However, the finding of NSC or frameshift variants shared between strains that originated from the same founder population irrespective of their disease status, for example, between a hypertensive strain and its respective normotensive control, indicates that they are unlikely to be responsible for disease phenotypes and are more likely a reflection of shared ancestry. We therefore sought clusters of coevolved transcripts that were unique to strains selected for disease phenotypes. Such clusters would be unique to an individual disease strain and would not be found even in the respective control strains with which they share immediate common ancestors, minimizing the likelihood that the clusters have arisen because of shared ancestry.

A unique coevolutionary cluster was identified in each disease model, the largest of which was found in the GK strain (Tables 2 and S5A). We also identified clusters of transcripts that were uniquely mutated in all disease models derived from the same founder, but not in the respective control strains. For example, clusters of transcripts that were mutated were identified in Wistar-derived strains SHR, SHRSP, and GK, but not in control strain WKY (Table S5B), and in the SD-derived disease models SS and LH, but not in control strain SR, LL, and LN (Table S5C). Because these NSC variants were unique to or were shared between these disease models and are not present in the respective control strains, it is plausible that a proportion of these were coselected and coevolved throughout the selective inbreeding process and contribute causally to the disease phenotype.

The Milan hypertensive rat strain (MHS) was derived from an outbred Wistar colony along with the normotensive MNS control strain. Hypertension in MHS rats has been shown to be due to excess renal sodium reabsorption (Bianchi et al., 1986). We identified a cluster of 65 transcripts (47 genes) showing NSC sequence variants and a cluster of 5 transcripts (4 genes) showing frameshift coding variants in MHS (Tables S5D and S5E). Importantly, the NSC cluster contains the *Add1* gene encoding Alpha-adducin, in which the amino acid substitution F316Y has been identified as a cause of hypertension in MHS (Bianchi et al., 2005; Ferrandi et al., 2010). Polymorphisms in

Table 2. Coevolutionary Gene Clusters Derived from Nonsynonymous and Frameshift Variants in Disease Models

Strain	Number of Transcripts in NSC Variant Cluster[a]	Number of Transcripts in Frameshift Variant Cluster[a]
BBDP	63 (47)	16 (12)
FHH	59 (46)	4 (4)
GK	230 (164)	22 (22)
LH	16 (15)	3 (2)
MHS	65 (47)	5 (4)
SBH	129 (103)	12 (12)
SHR	12 (12)	2 (2)
SHRSP	51 (39)	3 (2)
SS	35 (17)	4 (2)

NSC variants, nonsynonymous coding variants. See also Figure S3 and Table S5.
[a]Genes shown in parentheses.

the human *ADD1* gene have also been associated with hypertension and responsiveness to antihypertensive medications in several populations (Cusi et al., 1997; Li, 2012). We also identified, along with *Add1*, genes *Slc4a2*, *Slc12a8*, and *Atp13a4*, which show NSC mutations in MHS. *Slc4a2* encodes the anion exchange protein 2, which has shown evidence for association with human hypertension (Sõber et al., 2009). *Slc12a8* belongs to the cation chloride cotransporter family (Hebert et al., 2004), and *Atp13a4* encodes a cation-transporting P-type ATPase (Kwasnicka-Crawford et al., 2005). In light of longstanding observations on the relationship between ion transport and hypertension in rats and humans (Bianchi et al., 1990; Lifton et al., 2001), the physiological consequences of mutations in these ion transporters merit further investigation.

The BBDP rat strain is an extensively studied model of type 1 diabetes, occurring in association with profound T cell lymphopenia with severe reduction or absence of CD4 and CD8 T cells (Jackson et al., 1983). We identified a cluster of 63 transcripts (47 genes) showing NSC variants and also 16 transcripts (12 genes) showing frameshift variants that are unique to BBDP (Tables S5F and S5G). The frameshifts include a 1 base pair (bp) deletion in *Gimap5*, a member of the GTPase of the immunity-associated protein family. This frameshift mutation was previously identified as the cause of T cell lymphopenia in BBDP (Dalberg et al., 2007; Hornum et al., 2002; MacMurray et al., 2002). The finding that two genes identified previously as disease susceptibility genes in MHS and BBDP were among the small gene sets that we defined as uniquely variant in these strains using coevolutionary cluster analysis suggests that genes underlying other QTLs are likely to be found within the coevolved gene sets of these and other disease strains and that these gene sets should be prioritized in future investigations of disease phenotypes in these strains.

Although we identified clusters that were shared between disease models derived from different founder colonies such as BBDP and MHS (n = 17 transcripts) or GK and SBH (n = 17 transcripts), no clusters were found that revealed potentially functional mutations across all models of hypertension, diabetes, or insulin resistance. Surprisingly, clusters were not found even in rat strains showing spontaneous hypertension (FHH, MHS, SHR, SHRSP, and LH) or salt-induced hypertension (SS and SBH), most likely reflecting heterogeneity in hypertension and related phenotypes among these rat strains.

Next, we investigated whether the human orthologs of rat genes either shared between or uniquely mutated in rat disease models had been associated in human genome-wide association studies (GWAS) for hypertension, metabolic phenotypes, or their complications. We found that such genes were significantly overrepresented in GWAS hits for hypertension or metabolism-related phenotypes (Fisher's exact test p value = 10^{-4}), suggesting that genes represented in clusters containing unique NSC mutations in disease models had not only coevolved, but were also functionally related and may also contribute to these or related disease phenotypes in humans.

Identification of Artificial Selective Sweeps

To identify selective sweeps that may have arisen during phenotype-driven selective breeding, we identified genomic regions that were either unique to or shared between multiple strains. At a p value of less than 10^{-5} (corresponding to FDR < 0.1%), 15,859 separate genomic segments were significantly shared between two or more strains or were unique to a particular strain, with segment sizes ranging from 20 kb to 2.9 Mb (Table S6). Moreover, of these 15,859 regions, 189 were unique to a single rat strain, of which 96 were unique to one of the 11 models of cardiovascular or metabolic disease. Examples of genomic segments that were present uniquely in FHH and SS, but not in any other strains, including their respective control strains, are shown in Figure 3A. Of all SNVs that were unique to a single strain, 50% reside within the 189 segments that were unique to a single strain. Because these 189 segments occupy only 0.8% of the genome, these data indicate that private SNVs are not randomly distributed throughout the genome but instead are highly concentrated in a small number of discrete regions of the genome. We hypothesize that private SNVs that are unique to a single strain reside within these regions because many of these regions were positively selected in the initial phenotype-driven derivation of these strains. These regions may therefore be enriched for selective sweeps. We term these regions putative artificial selective sweep (PASS) regions.

Because regions under selection pressure tend to have elevated linkage disequilibrium (LD) (Xia et al., 2009), we estimated the pairwise LD between all of the SNVs within the detected PASS regions. Across the whole genome, strong LD was observed between adjacent pairs of SNVs. LD decay was slow and r^2 reduced to 50% of maximum at a mean distance of ~11 kb, although average r^2 was greater than 0.2 even at a distance of 0.5 Mb (Figure 3B). Within PASS regions, however, there was almost no decay of LD, and at a distance of 0.5 Mb, r^2 was 4-fold higher in PASS regions than the genome-wide average (Figure 3B). This strong LD completely disappeared after permutation of the SNVs (Figure S4). The increased LD in these regions validates the method used to detect PASS regions and is consistent with the hypothesis that these regions were under positive selection pressure during the original derivation of these strains.

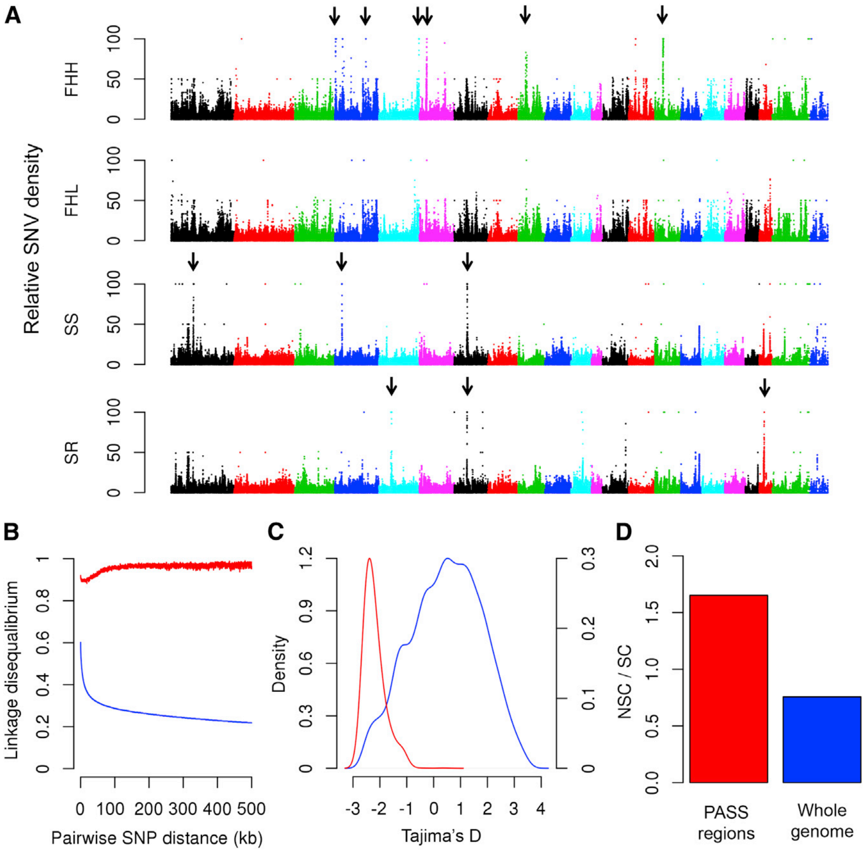

Figure 3. Examples of Putative Artificial Selective Sweep Regions Identified in Laboratory Rat Strains

(A) Relative SNV density (RSD) in 10 kb windows for four rat strains plotted along rat chromosomes separated by colors. Black arrows indicate putative artificial selective sweep (PASS) regions identified in respective rat strains. Closely adjacent but distinct PASS regions on the same chromosome are represented by a single arrow. The PASS region on chromosome 7 in SS is adjacent to but not overlapping with the chromosome 7 PASS region in SR. (For detail, see Table S6).

(B) Average linkage disequilibrium (LD) between pairs of SNVs within a distance ranging from 1 bp to 0.5 Mb. The blue line represents average LD at whole-genome level; the red line represents LD in PASS regions.

(C) Distribution of Tajima's D in 10 kb windows in the entire genome (blue line) and in PASS regions (red line).

(D) Ratio of nonsynonymous coding (NSC) variants to synonymous coding (SC) variants at genome level (blue bar) and in PASS regions (red bar).

See also Figure S4 and Tables S6 and S7.

To assess whether the PASS regions identified by the RSD analysis were due to the varying demographic history of the rat strains or, alternatively, were due to the effects of selection pressures exerted during derivation of the strains, we performed coalescent simulations that accounted for the known demographic history of the 22 laboratory rat strains, but not for the selection pressure exerted on them. In our null model, obtained by simulation on demographic history without phenotypic selection, no genomic region unique to a strain with length greater than 40 kb was observed, with a statistically unlikely probability of observing unique haplotype blocks of length 40 kb ($p = 0.0003$) or 30 kb ($p = 0.002$). All of the PASS regions that we identified were longer than or equal to 30 kb, and some were more than 1 Mb (Table S6). The identification of PASS regions that were considerably longer than those observed under the null model without selection provides evidence that PASS regions are enriched for positively selected haplotype blocks.

As further evidence that the identified PASS regions were positively selected during the phenotype-driven derivation of these strains, we estimated the population genetics parameter Tajima's D in each 10 kb window used for identification of PASS regions. FDR was calculated using the null distribution of Tajima's D derived from the coalescent simulation data. PASS regions showed highly negative Tajima's D values (Figure 3C), with more than 70% of the 10 kb windows within the PASS regions showing Tajima's D less than −2.08 (FDR $\leq$ 0.05).

Functional Significance of Selective Sweeps

As an initial test of whether the identified PASS regions are of functional significance, we estimated the ratio of NSC variants to SC variants in PASS regions. PASS regions were highly enriched for NSC mutations as compared to the rest of the genome, with an NSC to SC ratio of 1.65 and 0.76, respectively, in PASS regions and across the whole genome (Fisher's exact test p value = 2.19×10^{-5}; Figure 3D).

We next assessed what proportion of PASS regions colocalized with known physiological quantitative trait loci (pQTLs) for the metabolic and cardiovascular phenotypes manifested in these strains. Of the 96 PASS regions that were unique to 1 of the 11 cardio-metabolic disease strains, 25 were colocalized with pQTLs reported in RGD (http://rgd.mcw.edu/) in the same strain in which each PASS region was identified. This overlap was marginally significant compared to the overlap expected by chance (permutation test p value = 0.04). Interestingly, the majority (78%) of the PASS regions were localized either directly under or in close proximity (~10 Mb) to a peak of at least one QTL linkage (Table S7).

To identify genes and pathways that may have contributed to phenotype-driven selection during generation of disease strains, we carried out gene ontology (GO) and KEGG pathway analyses of genes in the PASS regions in these strains. Only two strains, SS and FHH, showed significant enrichment ($p < 0.05$) for specific biological processes or pathways. In FHH, 44 genes reside within 14 FHH-specific PASS regions (Figure 3A), and these were enriched for genes involved in the renin-angiotensin system (RAS) and in proteolysis ($p = 3.4 \times 10^{-4}$ and 1.6×10^{-8}, respectively).

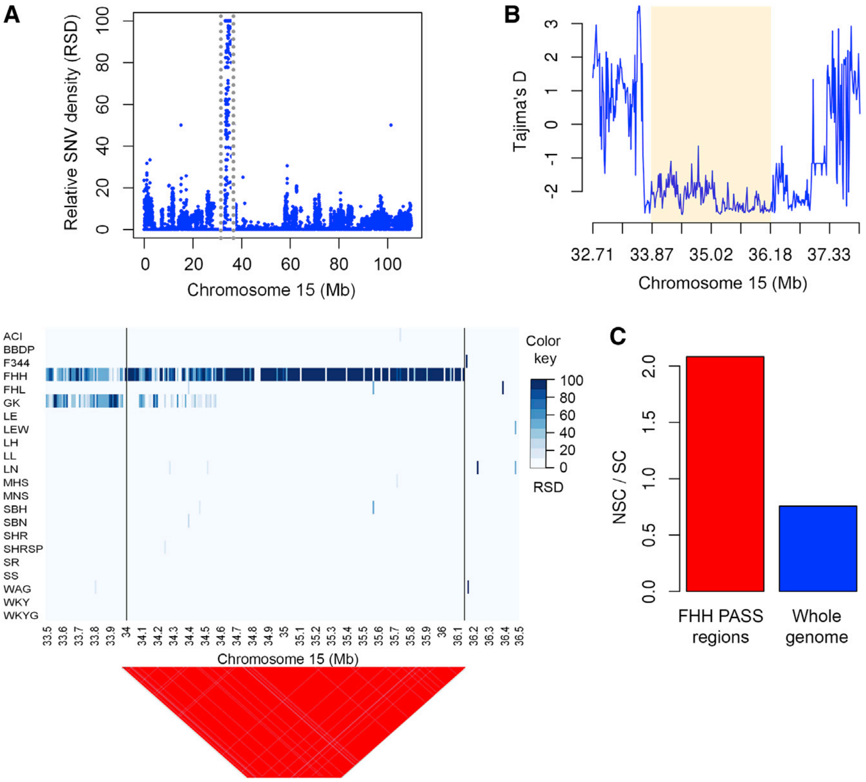

Figure 4. FHH PASS Regions on Chromosome 15

(A) Blue dots represent relative SNV density (RSD) in 10 kb windows for the FHH rat strain on chromosome 15. The genomic region unique to the FHH strain is highlighted by the gray dotted lines. Heatmap (bottom) showing RSD in the genomic region unique to FHH and flanking regions across all laboratory rat strains sequenced. Only the FHH rat strain showed an RSD value equal to 100. Remaining strains showed an RSD value of zero across almost all of the PASS regions, indicating that only the FHH strain shows SNVs against the BN reference genome in this region. Linkage disequilibrium (LD) structure of SNVs in the genomic region unique to FHH show that ~98% of the SNVs in this region were in significant LD.

(B)Tajima's D in genomic region unique to FHH rat strain. The PASS region unique to FHH is highlighted, showing highly negative Tajima's D value.

(C) The genomic region unique to FHH shows a markedly increased ratio of NSC to SC variants.

Three genes within FHH PASS regions were components of RAS, including *Cma1* and *CtsG*, both of which are reported to mediate non-angiotensin conversion enzyme (ACE)-dependent conversion of angiotensin I to angiotensin II (Uehara et al., 2013). The RAS system is central to the regulation of blood pressure in all mammals, and FHH rats show reduced sensitivity to angiotensin II, which has been proposed as a cause of FHH susceptibility to renal disease (van Rodijnen et al., 2002). In humans, a haplotype at *CMA1* has been weakly associated ($p = 2 \times 10^{-4}$) with hypertension in Han Chinese (Wu et al., 2012), and in mice, chymase 1 has been proposed to modulate regulation of blood pressure by angiotensin II (Li et al., 2004). In addition, ACE inhibitors and angiotensin receptor blockers are among the most widely used antihypertensive agents (Kobori et al., 2007). *Cma1* and *Ctsg* have NSC sequence variants that are unique to FHH among rat strains (His→Arg in *Cma1*; Ala→Thr in *Ctsg*). The histidine at codon 94 in *Cma1* is conserved in all mammals. *Cma1* and *Ctsg* are therefore compelling candidates as regulators of blood pressure in FHH and, because of their enzymatic function in non-ACE-dependent conversion of angiotensin I to angiotensin II, are potential novel antihypertensive drug targets.

The 44 genes in FHH-specific PASS regions have a markedly increased NSC-to-SC ratio (Figure 4C) and are highly enriched for GO biological processes related to proteolysis. The 12 genes in this GO category are all mast cell proteases or belong to the family of granzymes, which are also expressed in mast cells and reside within five FHH PASS regions on a 2.13 Mb segment of chromosome (Chr) 15 that shows highly negative Tajima's D (Figures 4A and 4B). Ten of these genes have NSC variants that are unique to FHH. FHH rats develop glomerulosclerosis and interstitial fibrosis with glomerular hyperfiltration, albuminuria, and end-stage renal failure (de Keijzer et al., 1988; Kreisberg and Karnovsky, 1978; Kriz et al., 1998). A consomic study in which FHH chromosomes were substituted with BN chromosomes showed that substitution of Chr 15 resulted in reduced blood pressure, albuminuria, and glomerular damage, indicating that genetic determinants of these phenotypes reside on FHH Chr 15 (Mattson et al., 2007). Mast cell numbers are increased in human hypertensive nephropathy and are associated with renal disease in the context of nephrectomy-induced hyperfiltration (Welker et al., 2008). Among the granzymes and mast cell proteases within the Chr 15 PASS regions, *GzmF* and *Mcpt10* contain nonconservative amino acid substitutions (Lys→Gln and Gly→Cys, respectively). Taken together, these data implicate this segment of Chr 15 and specifically the unique FHH variation in granzymes and mast cell proteases in the development of hypertension-induced renal damage in this rat strain.

In SS, 16 protein-coding genes reside within 4 SS-specific PASS regions (Figure 3A and Table S6), and these were enriched for aromatic compound catabolic processes ($p = 3.9 \times 10^{-5}$), owing to the presence of the three paraoxonase genes *Pon1*, *Pon2*, and *Pon3*, in one of the SS PASS regions on Chr 4. Paraoxonase genes protect against oxidative stress and have been implicated in atherosclerosis and diabetes mellitus in humans (Précourt et al., 2011) and blood pressure regulation in mice (Gamliel-Lazarovich et al., 2012). Oxidative stress is associated with endothelial dysfunction and hypertension in humans and in a wide range of experimental models, including the Dahl SS rat (Kushiro et al., 2005; Swei et al., 1997; Vaziri and Rodríguez-Iturbe, 2006). Because the paraoxonase genes overlap with SS QTLs for hypertension (Table S7), the paraoxonase

genes are compelling candidates as contributors to the hypertension phenotype in the SS strain.

DISCUSSION

Laboratory rat strains have been studied extensively for cardiovascular and metabolic phenotypes for several decades, but the vast majority of the genes and mutations underlying these phenotypes remain to be identified. This is at least in part because of the absence of the genome sequences and the lack of comprehensive catalogs of genomic variants for these strains. Previous catalogs of variants in the rat were restricted to 20,238 SNVs in 167 inbred rat strains, which have been used mostly as markers for QTL mapping (Saar et al., 2008). More recently, the report of 3.6 million SNVs and 0.3 million indels in the SHR strain has facilitated research aimed at understanding the mechanisms underlying complex traits in this strain (Atanur et al., 2010; Heinig et al., 2010; McDermott-Roe et al., 2011; Simonis et al., 2012). Another constraint has been that existing data sets of rat variants are biased toward single-nucleotide changes, and apart from SHR, other types of variants such as indels were partially or completely missing. This study reports an extensive catalog of 9.6 million SNVs, 3.5 million short indels, and 719,929 structural variants across 27 laboratory rat strains that are among the most widely used animal models of hypertension and diabetes.

We used ~2 Mb of high-quality BAC sequence from the nonreference strain LE/Stm to estimate the false-positive and false-negative rates of variant calling. Estimates of false positive and false negatives reported in other model organisms have been based on only the accessible part of the genome (Keane et al., 2011), which may underestimate the false-negative rate at the level of the whole genome. Although we estimate relatively high false-negative rates (8.1% and 10.98% for SNVs and indels, respectively), these false-negative rates are lower than those reported for mouse genome sequences (Keane et al., 2011) and represent upper estimates for missing variants as measured against the entire reference genome, rather than just the accessible genome. The false-positive rates were also lower in our sequences than those reported by Keane et al. for the mouse. The reasons for the lower false-positive and -negative rates may relate to the longer sequence reads and improved algorithms for mapping and variant calling used in the present studies.

We used the new sequence data from the 27 rat strains for two main purposes: to provide a firm foundation for understanding the phylogenetic history of the laboratory rat and to give new insights into the genetic and pathophysiological basis of the disease phenotypes for which the various rat strains were selected. Our phylogenetic analysis indicates that differences in laboratory rat strains are primarily due to genetic differences in the founder colonies from which the rat strains were derived, reflecting differences that were most likely present in the original outbred founder populations (Figure 1).

We used two approaches to give new insights into the genetic and pathophysiological basis of disease phenotypes in rat strains. First, we used the phylogenetic history of the strains to identify clusters of genes with correlated evolutionary rates. Such methods have been widely used to identify coevolving and functionally related proteins and small RNA pathways (de Juan et al., 2013; Pagliarini et al., 2008; Pazos and Valencia, 2001; Tabach et al., 2013). We defined coevolutionary clusters that were unique to each disease strain and among these clusters of genes identified two genes, *Add1* and *Gimap5*, previously shown by positional cloning to underlie susceptibility to hypertension in MHS and lymphopenia and diabetes in BBDP, respectively (Bianchi et al., 2005; Ferrandi et al., 2010; Hornum et al., 2002; MacMurray et al., 2002). Given the small number of genes shown to underlie rat disease phenotypes (Aitman et al., 2008), the finding of *Add1* in MHS and *Gimap5* in BBDP in coevolutionary clusters for these strains provides proof of concept of the efficacy of this approach in these rat strains. We also found among the coevolutionary clusters for MHS three further ion transport genes (*Slc4a2*, *Slc12a8*, *Atp13a4*), of which one, *Slc4a2*, shows a predicted nonconservative Gly→Asp amino acid substitution. The longstanding relationship between dysregulated ion transport and hypertension in MHS rats and in humans (Bianchi et al., 1990; Lifton et al., 2001) suggests these ion transporters as compelling candidates as hypertension susceptibility genes. Given that the human orthologs of rat genes within coevolutionary clusters showed significant enrichment for GWAS hits for hypertension and metabolism-related phenotypes, further study of these genes may shed light on the pathogenetic mechanisms for these phenotypes in both species.

Our second approach to gaining new insights into disease phenotypes was to identify putative selective sweeps, which we term PASS regions, that were unique to individual strains and may have been retained during the derivation of these disease models. Precedents for this type of analysis exist in the study of domestication of other species, including maize, silkworm, chicken, and dog (Axelsson et al., 2013; Rubin et al., 2010; Wright et al., 2005; Xia et al., 2009), but the approach has not been applied previously to the study of disease phenotypes. Our analyses took account of the known demographic history of the rat strains and used several methods to distinguish genomic segments that arose due to selection from those that may have arisen from population structure.

First, the PASS regions that we identified as unique to individual rat strains ranged in length from 30 kb to 1.57 Mb, whereas a null model obtained by coalescent simulation that took into account demographic history identified no regions with length > 30 kb. Second, we showed that LD was markedly increased across PASS regions compared to the rest of the genome. Third, we showed highly negative Tajima's D values within PASS regions, strongly supporting the presence of true selective sweeps among the identified PASS regions. Finally, we found that the ratio of nonsynonymous-to-synonymous coding variants in PASS regions was more than double that across the rest of the genome (1.65 and 0.76, respectively). These various approaches, used as indicators of evolutionary selection in a variety of scenarios (Axelsson et al., 2013; Rubin et al., 2010; Wright et al., 2005; Xia et al., 2009), strongly support the view that the identified PASS regions are significantly enriched for genomic segments retained by phenotype selection during derivation of these strains.

The PASS regions that we identified were significantly enriched for cardiovascular or metabolic QTLs previously mapped in these strains. In addition, where there was overlap between QTLs and PASS regions, the majority of PASS regions were localized at or close to the peak of the QTL linkage, in most cases suggesting candidates for these QTLs and in some cases single genes. These genes may now be tested in targeted gene knockin or knockout experiments that may now be easily performed in the rat on any genetic background (Jacob et al., 2010).

To identify specific pathways that may have contributed to phenotype-driven selection during derivation of disease strains, we carried out GO analyses of genes within PASS regions and identified enrichment for specific biological processes in two strains, FHH and SS. PASS regions in FHH showed enrichment for genes in the RAS and for proteolytic enzymes. The finding of enrichment for genes in the RAS is not unexpected, given the central role of this system in blood pressure regulation. However, canonical RAS genes that are either hypertension susceptibility genes or drug targets are related to ACE, whereas two of the genes in the FHH PASS regions mediate non-ACE dependent conversion of angiotensin I to angiotensin II. These genes, *Cma1* and *Ctsg*, are therefore compelling hypertension candidate genes, particularly as the histidine at codon 94 in *Cma1* that is uniquely variant in FHH among all rat strains is conserved in all mammals. The finding in FHH PASS regions of enrichment for mast cell proteins, some of which show NSC variants at highly conserved amino acids, suggests a previously unidentified pathway for hypertension-induced nephropathy that is consistent with previous observations in human and rat hypertension, the mechanism for which is at present unknown. Similar considerations apply to the enrichment of genes associated with protection against oxidative stress, found to be enriched in PASS regions in SS.

We note that our analysis of PASS regions and coevolved clusters across disease models did not identify shared clusters of genes that were common to all strains that carry the same disease phenotype, suggesting differing genetic etiologies for complex traits across these disease models, as previously suggested (Stoll et al., 2000). This may reflect the differing pathophysiology of traits such as hypertension across these strains (Jong, 1984) but is also likely due, in part, to the different genetic backgrounds on which these models were developed.

The data from these studies, including the genome sequences, catalogs of sequence variants, sets of coevolved gene clusters, and PASS regions across 27 rat strains, represent a step change in the genome resources available for study of complex phenotypes in the rat model. Identification of genes underlying complex phenotypes in this model has until now rested on laborious and lengthy gene-mapping studies that have mostly localized disease genes to the genome at a resolution of tens of mega base pairs. By taking advantage of whole-genome sequences and population structure in 27 strains, these studies identify coevolved segments and haplotype blocks that are unique to individual disease strains. These genes and pathways, including those previously identified as QTL genes and unidentified genes such as non-ACE-dependent angiotensin converting enzymes, mast cell proteases, and proteins that protect against oxidative stress, were most likely highlighted in these studies because they were selected in the original phenotype-driven derivation of these strains. We believe this to be the first evolutionary analysis of artificial selection for disease phenotypes and suggest that further analysis of these genes will confirm many of them as new genes for these phenotypes that will shed new light on the pathogenesis of these conditions in rats and humans.

EXPERIMENTAL PROCEDURES

Also see Extended Experimental Procedures.

Rat Strains and Genome Sequencing

We sequenced the genomes of 25 rat strains on the Illumina sequencing platform (Table 1) and analyzed these together with two rat genomes that we sequenced previously.

Mapping to the Reference Genome

Quality filtered Illumina paired-end and/or mate pair reads were mapped to the BN reference genome RGSC-3.4 (Gibbs et al., 2004) using BWA-0.5.8c (Li and Durbin, 2009). The genome (BN.Lx) sequenced previously on the SOLiD sequencing platform was mapped using BFAST-0.6.4e (Homer et al., 2009).

Single-Nucleotide Variants and Short Indel Detection

Genomic variants (single-nucleotide variants and short indels [1–15 bp]) were detected using the Genome Analysis Toolkit (GATK version 1.0.6001) (DePristo et al., 2011; McKenna et al., 2010).

Structural Variant Prediction

To identify structural variants, we used two independent methods. Structural variants including insertions, deletions, inversions, and translocations were predicted using the software tool BreakDancer1.2 (Chen et al., 2009). In addition, large deletions were predicted using a custom Perl script as described previously (Atanur et al., 2010), using mapping flags provided by BWA for paired-end reads (Li and Durbin, 2009).

Functional Consequence Analysis of Predicted Variants

Functional consequences of predicted variants (SNVs and short indels) were estimated using "variant effect predictor (VEP)-v2.4" (McLaren et al., 2010) on the ENSEMBL 66 gene set.

Validation of Genomic Variants

To estimate sensitivity and specificity of variant calls, the sequence of 13 bacterial artificial chromosome (BAC) clones from various chromosomes (~2 Mb in length) of LE/Stm rat were downloaded from the EMBL web site (accession numbers FO117624- FO117632 and FO181540- FO181543).

Reconstruction of the Phylogenetic Tree

A distance matrix, derived from SNVs between all possible pairs of strains, was used to construct the phylogenetic tree using the Fitch-Margoliash method with contemporary tips, version 3.69 from package Phylip (Felsenstein, 1989), with 1,000 bootstraps.

Identification of Coevolutionary Gene Clusters

Coevolutionary gene clusters were identified using a mirror tree approach. We used two independent methods to correct for population structure: (1) principal component analysis and (2) a linear mixture model.

Identification of Selective Sweeps between Different Rat Strains

To identify genomic regions shared between different rat strains, relative SNV density (RSD) in nonoverlapping 10 kb windows was calculated in all 26 rat strains (excluding BN.Lx) using the following formula:

$$RSDi = \frac{\text{Number of SNVs in strain } i}{\text{Total number of SNVs}} \; x \; 100$$

Chi-square statistics were used to determine the goodness of fit to a model of equal distribution of SNVs within each 10 kb window between "n" strains. Chi-square statistics were calculated for all possible combinations of strains according to:

$$SC = \min\left(\binom{n}{k = 1 \ldots n} \sum_{i=1}^{k} \frac{(O_i - E_i)^2}{E_i}\right)$$

Adjacent 10 kb windows were merged if they were shared between the same strain combinations.

Calculation of Linkage Distribution

Linkage distribution between the SNVs within the coevolutionary gene clusters and SNVs within PASS regions was calculated using Haploview 4 (Barrett et al., 2005).

Coalescent Simulations

Coalescent simulations were performed using the program msms (Ewing and Hermisson, 2010).

Calculation of Population Genetics Statistics Tajima's D

Tajima's D was calculated in nonoverlapping 10 kb windows using custom Perl script as described in (Tajima, 1989). FDR was calculated using the null distribution of Tajima's D from simulated data.

ACCESSION NUMBERS

All sequence reads from this study are deposited in the EBI Sequence Read Archive under accession number ERP002160. All sequence variants are deposited in RGD (http://rgd.mcw.edu/).

SUPPLEMENTAL INFORMATION

Supplemental Information includes Extended Experimental Procedures, four figures, and eight tables and can be found with this article online at http://dx.doi.org/10.1016/j.cell.2013.06.040.

ACKNOWLEDGMENTS

T.J.A. acknowledges funding from the Medical Research Council, British Heart Foundation Center of Research Excellence (RE/08/002), and the EU-funded Euratrans project (HEALTH-F4-2010-241504). We thank the National BioResource Project-Rat (http://www.anim.med.kyoto-u.ac.jp/NBR/) for providing DNA from rat strain LE/Stm and Jean Sassard for provision of rats for DNA sequencing of LH, LN, and LL rats. E.C. is supported by a TOP grant (700.58.303) from the Netherlands Research Council (NWO-CW). H.J. acknowledges funding from NIH grant 5R01HL069321. N.H. acknowledges funding from Euratrans and the BMPF-funded German Center for Cardiovascular Research. D.G. acknowledges support from a Wellcome Trust Senior Fellowship in Basic Biomedical Science (057733), a Wellcome Trust Core Award Grant (075491/Z/04), and support from the Institute of Cardiometabolism and Nutrition (ICAN, ANR-10-IAHU-05). M.R.G. is supported by NIH/NHLBI HL094446 and Robert M. Hearin Foundation. P.F. acknowledges funding from EMBL and from the EU-funded Euratrans project. We thank Allen Cowley for helpful discussions on the manuscript.

Received: January 25, 2013
Revised: April 30, 2013
Accepted: June 21, 2013
Published: July 25, 2013

REFERENCES

Aitman, T.J., Critser, J.K., Cuppen, E., Dominiczak, A., Fernandez-Suarez, X.M., Flint, J., Gauguier, D., Geurts, A.M., Gould, M., Harris, P.C., et al. (2008). Progress and prospects in rat genetics: a community view. Nat. Genet. *40*, 516–522.

Atanur, S.S., Birol, I., Guryev, V., Hirst, M., Hummel, O., Morrissey, C., Behmoaras, J., Fernandez-Suarez, X.M., Johnson, M.D., McLaren, W.M., et al. (2010). The genome sequence of the spontaneously hypertensive rat: Analysis and functional significance. Genome Res. *20*, 791–803.

Axelsson, E., Ratnakumar, A., Arendt, M.L., Maqbool, K., Webster, M.T., Perloski, M., Liberg, O., Arnemo, J.M., Hedhammar, A., and Lindblad-Toh, K. (2013). The genomic signature of dog domestication reveals adaptation to a starch-rich diet. Nature *495*, 360–364.

Barrett, J.C., Fry, B., Maller, J., and Daly, M.J. (2005). Haploview: analysis and visualization of LD and haplotype maps. Bioinformatics *21*, 263–265.

Bianchi, G., Ferrari, P., Salvati, P., Salardi, S., Parenti, P., Cusi, D., and Guidi, E. (1986). A renal abnormality in the Milan hypertensive strain of rats and in humans predisposed to essential hypertension. J. Hypertens. Suppl. *4*, S33–S36.

Bianchi, G., Ferrari, P., Cusi, D., Tripodi, G., and Barber, B. (1990). Genetic aspects of ion transport systems in hypertension. J. Hypertens. Suppl. *8*, S213–S218.

Bianchi, G., Ferrari, P., and Staessen, J.A. (2005). Adducin polymorphism: detection and impact on hypertension and related disorders. Hypertension *45*, 331–340.

Chen, K., Wallis, J.W., McLellan, M.D., Larson, D.E., Kalicki, J.M., Pohl, C.S., McGrath, S.D., Wendl, M.C., Zhang, Q., Locke, D.P., et al. (2009). BreakDancer: an algorithm for high-resolution mapping of genomic structural variation. Nat. Methods *6*, 677–681.

Cusi, D., Barlassina, C., Azzani, T., Casari, G., Citterio, L., Devoto, M., Glorioso, N., Lanzani, C., Manunta, P., Righetti, M., et al. (1997). Polymorphisms of alpha-adducin and salt sensitivity in patients with essential hypertension. Lancet *349*, 1353–1357.

Dalberg, U., Markholst, H., and Hornum, L. (2007). Both Gimap5 and the diabetogenic BBDP allele of Gimap5 induce apoptosis in T cells. Int. Immunol. *19*, 447–453.

de Juan, D., Pazos, F., and Valencia, A. (2013). Emerging methods in protein co-evolution. Nat. Rev. Genet. *14*, 249–261.

de Keijzer, M.H., Provoost, A.P., and Molenaar, J.C. (1988). Glomerular hyperfiltration in hypertensive fawn-hooded rats. Ren. Physiol. Biochem. *11*, 103–108.

DePristo, M.A., Banks, E., Poplin, R., Garimella, K.V., Maguire, J.R., Hartl, C., Philippakis, A.A., del Angel, G., Rivas, M.A., Hanna, M., et al. (2011). A framework for variation discovery and genotyping using next-generation DNA sequencing data. Nat. Genet. *43*, 491–498.

Doggrell, S.A., and Brown, L. (1998). Rat models of hypertension, cardiac hypertrophy and failure. Cardiovasc. Res. *39*, 89–105.

Ewing, G., and Hermisson, J. (2010). MSMS: a coalescent simulation program including recombination, demographic structure and selection at a single locus. Bioinformatics *26*, 2064–2065.

Felsenstein, J. (1989). PHYLIP - Phylogeny Inference Package (Version 3.2). Cladistics *5*, 164–166.

Ferrandi, M., Molinari, I., Torielli, L., Padoani, G., Salardi, S., Rastaldi, M.P., Ferrari, P., and Bianchi, G. (2010). Adducin- and ouabain-related gene variants predict the antihypertensive activity of rostafuroxin, part 1: experimental studies. Sci. Transl. Med. *2*, 59ra86.

Gamliel-Lazarovich, A., Abassi, Z., Khatib, S., Tavori, H., Vaya, J., Aviram, M., and Keidar, S. (2012). Paraoxonase1 deficiency in mice is associated with hypotension and increased levels of 5,6-epoxyeicosatrienoic acid. Atherosclerosis *222*, 92–98.

Gibbs, R.A., Weinstock, G.M., Metzker, M.L., Muzny, D.M., Sodergren, E.J., Scherer, S., Scott, G., Steffen, D., Worley, K.C., Burch, P.E., et al.; Rat Genome Sequencing Project Consortium. (2004). Genome sequence of the Brown Norway rat yields insights into mammalian evolution. Nature *428*, 493–521.

Hebert, S.C., Mount, D.B., and Gamba, G. (2004). Molecular physiology of cation-coupled Cl- cotransport: the SLC12 family. Pflugers Arch. *447*, 580–593.

Heinig, M., Petretto, E., Wallace, C., Bottolo, L., Rotival, M., Lu, H., Li, Y., Sarwar, R., Langley, S.R., Bauerfeind, A., et al.; Cardiogenics Consortium. (2010). A trans-acting locus regulates an anti-viral expression network and type 1 diabetes risk. Nature *467*, 460–464.

Homer, N., Merriman, B., and Nelson, S.F. (2009). BFAST: an alignment tool for large scale genome resequencing. PLoS ONE *4*, e7767.

Hornum, L., Rømer, J., and Markholst, H. (2002). The diabetes-prone BB rat carries a frameshift mutation in Ian4, a positional candidate of Iddm1. Diabetes *51*, 1972–1979.

Jackson, R., Kadison, P., Buse, J., Rassi, N., Jegasothy, B., and Eisenbarth, G.S. (1983). Lymphocyte abnormalities in the BB rat. Metabolism *32*(7, Suppl 1), 83–86.

Jacob, H.J., Lazar, J., Dwinell, M.R., Moreno, C., and Geurts, A.M. (2010). Gene targeting in the rat: advances and opportunities. Trends Genet. *26*, 510–518.

Jong, D.W. (1984). Handbook of Hypertension, *Volume 4* (Oxford: Elsevier).

Kang, H.M., Zaitlen, N.A., Wade, C.M., Kirby, A., Heckerman, D., Daly, M.J., and Eskin, E. (2008). Efficient control of population structure in model organism association mapping. Genetics *178*, 1709–1723.

Keane, T.M., Goodstadt, L., Danecek, P., White, M.A., Wong, K., Yalcin, B., Heger, A., Agam, A., Slater, G., Goodson, M., et al. (2011). Mouse genomic variation and its effect on phenotypes and gene regulation. Nature *477*, 289–294.

Kobori, H., Nangaku, M., Navar, L.G., and Nishiyama, A. (2007). The intrarenal renin-angiotensin system: from physiology to the pathobiology of hypertension and kidney disease. Pharmacol. Rev. *59*, 251–287.

Kreisberg, J.I., and Karnovsky, M.J. (1978). Focal glomerular sclerosis in the fawn-hooded rat. Am. J. Pathol. *92*, 637–652.

Kriz, W., Hosser, H., Hähnel, B., Gretz, N., and Provoost, A.P. (1998). From segmental glomerulosclerosis to total nephron degeneration and interstitial fibrosis: a histopathological study in rat models and human glomerulopathies. Nephrol. Dial. Transplant. *13*, 2781–2798.

Kurtz, T.W., Montano, M., Chan, L., and Kabra, P. (1989). Molecular evidence of genetic heterogeneity in Wistar-Kyoto rats: implications for research with the spontaneously hypertensive rat. Hypertension *13*, 188–192.

Kushiro, T., Fujita, H., Hisaki, R., Asai, T., Ichiyama, I., Kitahara, Y., Koike, M., Sugiura, H., Saito, F., Otsuka, Y., and Kanmatsuse, K. (2005). Oxidative stress in the Dahl salt-sensitive hypertensive rat. Clin. Exp. Hypertens. *27*, 9–15.

Kwasnicka-Crawford, D.A., Carson, A.R., Roberts, W., Summers, A.M., Rehnström, K., Järvelä, I., and Scherer, S.W. (2005). Characterization of a novel cation transporter ATPase gene (ATP13A4) interrupted by 3q25-q29 inversion in an individual with language delay. Genomics *86*, 182–194.

Li, Y.Y. (2012). α-Adducin Gly460Trp gene mutation and essential hypertension in a Chinese population: a meta-analysis including 10,960 subjects. PLoS ONE *7*, e30214.

Li, H., and Durbin, R. (2009). Fast and accurate short read alignment with Burrows-Wheeler transform. Bioinformatics *25*, 1754–1760.

Li, M., Liu, K., Michalicek, J., Angus, J.A., Hunt, J.E., Dell'Italia, L.J., Feneley, M.P., Graham, R.M., and Husain, A. (2004). Involvement of chymase-mediated angiotensin II generation in blood pressure regulation. J. Clin. Invest. *114*, 112–120.

Lifton, R.P., Gharavi, A.G., and Geller, D.S. (2001). Molecular mechanisms of human hypertension. Cell *104*, 545–556.

Lindsey, J.R. (1979). Historical foundations in the laboratory rat. In The Laboratory Rat, L. H.J, J.R. Baker and S.H. Weisbroth, eds. (New York: Academic Press), pp. 1–36.

MacMurray, A.J., Moralejo, D.H., Kwitek, A.E., Rutledge, E.A., Van Yserloo, B., Gohlke, P., Speros, S.J., Snyder, B., Schaefer, J., Bieg, S., et al. (2002). Lymphopenia in the BB rat model of type 1 diabetes is due to a mutation in a novel immune-associated nucleotide (Ian)-related gene. Genome Res. *12*, 1029–1039.

Mattson, D.L., Dwinell, M.R., Greene, A.S., Kwitek, A.E., Roman, R.J., Cowley, A.W., Jr., and Jacob, H.J. (2007). Chromosomal mapping of the genetic basis of hypertension and renal disease in FHH rats. Am. J. Physiol. Renal Physiol. *293*, F1905–F1914.

McDermott-Roe, C., Ye, J., Ahmed, R., Sun, X.M., Serafín, A., Ware, J., Bottolo, L., Muckett, P., Cañas, X., Zhang, J., et al. (2011). Endonuclease G is a novel determinant of cardiac hypertrophy and mitochondrial function. Nature *478*, 114–118.

McKenna, A., Hanna, M., Banks, E., Sivachenko, A., Cibulskis, K., Kernytsky, A., Garimella, K., Altshuler, D., Gabriel, S., Daly, M., and DePristo, M.A. (2010). The Genome Analysis Toolkit: a MapReduce framework for analyzing next-generation DNA sequencing data. Genome Res. *20*, 1297–1303.

McLaren, W., Pritchard, B., Rios, D., Chen, Y., Flicek, P., and Cunningham, F. (2010). Deriving the consequences of genomic variants with the Ensembl API and SNP Effect Predictor. Bioinformatics *26*, 2069–2070.

Pagliarini, D.J., Calvo, S.E., Chang, B., Sheth, S.A., Vafai, S.B., Ong, S.E., Walford, G.A., Sugiana, C., Boneh, A., Chen, W.K., et al. (2008). A mitochondrial protein compendium elucidates complex I disease biology. Cell *134*, 112–123.

Pazos, F., and Valencia, A. (2001). Similarity of phylogenetic trees as indicator of protein-protein interaction. Protein Eng. *14*, 609–614.

Précourt, L.P., Amre, D., Denis, M.C., Lavoie, J.C., Delvin, E., Seidman, E., and Levy, E. (2011). The three-gene paraoxonase family: physiologic roles, actions and regulation. Atherosclerosis *214*, 20–36.

Rapp, J.P. (2000). Genetic analysis of inherited hypertension in the rat. Physiol. Rev. *80*, 135–172.

Rubin, C.J., Zody, M.C., Eriksson, J., Meadows, J.R., Sherwood, E., Webster, M.T., Jiang, L., Ingman, M., Sharpe, T., Ka, S., et al. (2010). Whole-genome resequencing reveals loci under selection during chicken domestication. Nature *464*, 587–591.

Saar, K., Beck, A., Bihoreau, M.T., Birney, E., Brocklebank, D., Chen, Y., Cuppen, E., Demonchy, S., Dopazo, J., Flicek, P., et al.; STAR Consortium. (2008). SNP and haplotype mapping for genetic analysis in the rat. Nat. Genet. *40*, 560–566.

Sato, T., Yamanishi, Y., Kanehisa, M., and Toh, H. (2005). The inference of protein-protein interactions by co-evolutionary analysis is improved by excluding the information about the phylogenetic relationships. Bioinformatics *21*, 3482–3489.

Simonis, M., Atanur, S.S., Linsen, S., Guryev, V., Ruzius, F.P., Game, L., Lansu, N., de Bruijn, E., van Heesch, S., Jones, S.J., et al. (2012). Genetic basis of transcriptome differences between the founder strains of the rat HXB/BXH recombinant inbred panel. Genome Biol. *13*, r31.

Sõber, S., Org, E., Kepp, K., Juhanson, P., Eyheramendy, S., Gieger, C., Lichtner, P., Klopp, N., Veldre, G., Viigimaa, M., et al.; Kooperative Gesundheitsforschung in der Region Augsburg Study; HYPertension in ESTonia Study; MRC British Genetics of Hypertension Study. (2009). Targeting 160 candidate genes for blood pressure regulation with a genome-wide genotyping array. PLoS ONE *4*, e6034.

St Lezin, E., Griffin, K.A., Picken, M., Churchill, M.C., Churchill, P.C., Kurtz, T.W., Liu, W., Wang, N., Kren, V., Zidek, V., et al. (1999). Genetic isolation of a chromosome 1 region affecting susceptibility to hypertension-induced renal damage in the spontaneously hypertensive rat. Hypertension *34*, 187–191.

Stoll, M., Kwitek-Black, A.E., Cowley, A.W., Jr., Harris, E.L., Harrap, S.B., Krieger, J.E., Printz, M.P., Provoost, A.P., Sassard, J., and Jacob, H.J.

(2000). New target regions for human hypertension via comparative genomics. Genome Res. *10*, 473–482.

Swei, A., Lacy, F., DeLano, F.A., and Schmid-Schönbein, G.W. (1997). Oxidative stress in the Dahl hypertensive rat. Hypertension *30*, 1628–1633.

Tabach, Y., Billi, A.C., Hayes, G.D., Newman, M.A., Zuk, O., Gabel, H., Kamath, R., Yacoby, K., Chapman, B., Garcia, S.M., et al. (2013). Identification of small RNA pathway genes using patterns of phylogenetic conservation and divergence. Nature *493*, 694–698.

Tajima, F. (1989). Statistical method for testing the neutral mutation hypothesis by DNA polymorphism. Genetics *123*, 585–595.

Tillier, E.R., and Charlebois, R.L. (2009). The human protein coevolution network. Genome Res. *19*, 1861–1871.

Uehara, Y., Miura, S.I., Yahiro, E., and Saku, K. (2013). Non-ACE pathway-induced angiotensin II production. Curr. Pharm. Des., 3054–3059.

van Rodijnen, W.F., van Lambalgen, T.A., Tangelder, G.J., van Dokkum, R.P., Provoost, A.P., and ter Wee, P.M. (2002). Reduced reactivity of renal microvessels to pressure and angiotensin II in fawn-hooded rats. Hypertension *39*, 111–115.

Vaziri, N.D., and Rodríguez-Iturbe, B. (2006). Mechanisms of disease: oxidative stress and inflammation in the pathogenesis of hypertension. Nat. Clin. Pract. Nephrol. *2*, 582–593.

Welker, P., Krämer, S., Groneberg, D.A., Neumayer, H.H., Bachmann, S., Amann, K., and Peters, H. (2008). Increased mast cell number in human hypertensive nephropathy. Am. J. Physiol. Renal Physiol. *295*, F1103–F1109.

Wright, S.I., Bi, I.V., Schroeder, S.G., Yamasaki, M., Doebley, J.F., McMullen, M.D., and Gaut, B.S. (2005). The effects of artificial selection on the maize genome. Science *308*, 1310–1314.

Wu, Y., Yang, H., Yang, B., Yang, K., and Xiao, C. (2012). Association of polymorphisms in prolylcarboxypeptidase and chymase genes with essential hypertension in the Chinese Han population. J. Renin Angiotensin Aldosterone Syst. Published online June 7, 2012. http://dx.doi.org/10.1177/1470320312448949.

Xia, Q., Guo, Y., Zhang, Z., Li, D., Xuan, Z., Li, Z., Dai, F., Li, Y., Cheng, D., Li, R., et al. (2009). Complete resequencing of 40 genomes reveals domestication events and genes in silkworm (Bombyx). Science *326*, 433–436.

EXTENDED EXPERIMENTAL PROCEDURES

Rat Strains

We sequenced the genomes of 25 rat strains, namely ACI/EurMcwi, BBDP/Wor, F344/NCrl, FHH/EurMcwi, FHL/EurMcwi, GK/Ox, LE/Stm, LEW/Crl, LEW/NCrlBR, LH/MavRrrc, LN/MavRrrc, LL/MavRrrc, MHS/Gib, MNS/Gib, SBH/Ygl, SBN/Ygl, SHR/NHsd, SHRSP/Gla, SS/Jr, SS/JrHsdMcwi, SR/Jr, WAG/Rij, WKY/Gla, WKY/NCrl, WKY/NHsd on the Illumina sequencing platform (Table 1). We also included in our analysis the genomes of two rat strains SHR/OlaIpcv and BN.LX that we had sequenced previously on the Illumina and SOLiD sequencing platform respectively (Atanur et al., 2010; Simonis et al., 2012), taking the number of analyzed genomes to 27.

Genome Sequencing

For the genomes which were sequenced on the Illumina platform, we used the following protocol. Five micrograms of DNA was used to construct paired-end whole-genome libraries with 300-600 bp insert size. Genomic DNA was prepared from liver or spleen by standard phenol chloroform extraction followed by treatment with DNase free RNase or using the Maxwell 16 DNA Purification kit (Promega). DNA quality was assessed by spectrophotometry and gel electrophoresis before library construction. Genomic DNA was sheared using a Covaris S2 instrument (KBioscience, Herts, UK). Shearing efficiency was assessed by Qubit 2.0 fluorometer measurements (Life Technologies Ltd, Paisley, UK) and gel electrophoresis. The library was prepared by following the Illumina Genomic DNA sample prep kit protocol (Illumina Inc., Hayward, CA). Constructed libraries were assessed with an Agilent 2100 Bioanalyser (Agilent Technologies, Edinburgh, UK) and quantified using a KAPA Illumina SYBR Universal Lib QPCR kit (Anachem Ltd, Bedfordshire, UK). The resulting libraries were sequenced on an Illumina HiSeq2000 following the manufacturer's instructions. We also sequenced the genome of BN/Mcwi, the rat strain from which the reference BN rat genome was derived, on the SOLiD sequencing platform, using protocols previously described (Simonis et al., 2012), to identify and exclude potential sequencing errors in the reference genome.

Mapping to the Reference Genome

Quality filtered Illumina paired-end and/or mate pair reads were mapped to the BN reference genome RGSC-3.4 (Gibbs et al., 2004) using the read alignment software Burrows-Wheeler Aligner (BWA-0.5.8c) (Li and Durbin, 2009). All default parameters were used while mapping reads using BWA except for the read trimming parameter. The reads were trimmed at the end if the phred scaled base quality score dropped below 20. Genomes sequenced on the SOLiD sequencing platform were mapped to the reference BN genome using BFAST-0.6.4e (Homer et al., 2009).

Single-Nucleotide Variants and Short Indel Detection

Genomic variants (SNV and short indels (1-15bp)) were detected using the Genome Analysis Toolkit (GATK version 1.0.6001) (DePristo et al., 2011; McKenna et al., 2010). Before calling variants, data were processed using various preprocessing steps. i) Clonal reads were masked independently for each library of every rat strain using Picard tools (http://sourceforge.net/projects/picard/) ii) Reads were realigned around the indels, by first identifying regions for realignment where at least one read contains an indel with a cluster of mismatching bases around it. iii) base quality scores were recalibrated using GATK, which provides empirically accurate base quality scores for each base in every read, while also correcting for error covariates like machine cycle and dinucleotide context as well as supporting platform-specific error covariates like color-space mismatches for the SOLiD platform (DePristo et al., 2011).

Reads mapped with mapping quality greater than or equal to 10 and bases with base quality greater than or equal to 17 were used for variant calling to avoid false positives due to errors in read alignment and sequencing errors.

After calling SNVs and indels we applied variant quality recalibration to exclude potential false positive variant calls. Given the set of known SNVs and the SNV annotations, soft filtering using the variant quality score recalibration function of GATK employs a variational Bayes Gaussian mixture model (GMM) to estimate the probability that each called variant is a true variant in the samples rather than a sequencer, alignment or data processing artifact (DePristo et al., 2011). As dbSNP125 (Sherry et al., 2001) contains only 35,186 SNVs in rat strains, the model was trained using high quality SNVs extracted from this data set. The top 30% of high quality SNVs were used as the training set for GMM.

Structural Variant Prediction

Structural variants, including large deletions, insertions, inversions and translocations, were predicted using the software tool BreakDancer1.2 (Chen et al., 2009). Only reads mapped with mapping quality more than 20 were used to call structural variants. In addition large deletions were predicted using custom Perl script using mapping flags provided by BWA for paired end reads (Li and Durbin, 2009). Read pairs mapped in forward- reverse (FR) orientation and flagged as improper pairs by BWA were extracted. Clusters of at least 2 improper pairs were identified where at least one end was mapped with quality greater than or equal to 20 and deletions were predicted using a custom Perl script. Further deletions were filtered if they spanned less than 100 bp on both ends of genomic gaps. Validation of large deletions using sequence data derived for the SHR/OlaIpcv strain and the above algorithms was previously ascertained at greater than 90% (Atanur et al., 2010).

Functional Consequence Analysis of Predicted Variants

Functional consequences of predicted variants (SNVs and short indels) were analyzed using a "variant effect predictor (VEP) – version 2.4" Perl script (McLaren et al., 2010), which is based on the ENSEMBL application programming interface (APIs). For functional consequences analysis the ENSEMBL 66 gene set was used.

Validation of Genomic Variants

To estimate sensitivity and specificity of variant calls, the sequence of 13 bacterial artificial chromosome (BAC) clones from various chromosomes (~2Mb in length) of LE/Stm rat were downloaded from the EMBL web site (accession numbers FO117624- FO117632 and FO181540- FO181543). 100 bp paired end reads were then generated from the BAC sequences and mapped back to the BN reference genome using BWA (Li and Durbin, 2009). SNVs and indels were called using GATK (DePristo et al., 2011; McKenna et al., 2010); 3,382 SNVs and 1,211 indels were called between LE/Stm BAC sequences and BN reference genome.

Accuracy was calculated as:

$$\text{Accuracy} = \frac{(\text{TP} + \text{TN})}{(\text{TP} + \text{TN} + \text{FP} + \text{FN})}$$

Where, TP = True positive, FP = False positive, TN = True negative, FN = False negative
Genomic variants in genes within putative artificial sweeps that were highlighted by gene ontology analysis and contained nonsynonymous coding variants (*Cma1*, *Ctsg*, *Mcpt10*, *Gzmf*) were confirmed either by capillary sequencing or by analysis of RNA-seq.

Reconstruction of the Phylogenetic Tree

To construct the phylogenetic tree, SNVs between all possible pairs of strains were identified. Distance between any pair of strains was calculated using the following formula:

$$\text{Distance} = \frac{\text{Number of SNVs between the pair of strains}}{\text{length of rat genome}}$$

A distance matrix was then used to construct the phylogenetic tree using the Fitch-Margoliash method with contemporary tips, version 3.69 from package Phylip (Felsenstein, 1989) with 1000 bootstraps.

Identification of Coevolutionary Gene Clusters

Coevolutionary gene clusters were identified using transcripts that showed nonsynonymous coding (NSC) mutations in at least one rat strain compared to the reference BN genome. For each transcript showing NSC variants, the evolutionary rate was calculated in each rat strain by dividing the number of NSC variants by the number of synonymous coding (SC) variants, both normalized by length of the transcript. Thus, an m x n matrix of evolutionary rate was obtained, where m represents the number of strains and n represents the number of transcripts (the evolutionary rate being zero in the reference BN genome). BN.Lx was excluded from this analysis because BN.Lx show a very small number of SNVs in comparison with the BN reference genome. We also separately identified coevolutionary clusters for indels resulting in a frameshift in transcript sequence by counting the number of indels causing a frameshift and normalizing this to transcript length. The analysis was carried out independently for NSC variants and frameshift variants.

To take into account the phylogenetic relationships (population structure) between the strains under consideration in the coevolutionary cluster analysis, we identified major components responsible for variability between strains using principal component analysis, and adjusted the evolutionary rate for these components prior to coevolutionary gene cluster analysis. A kinship matrix derived from SNVs between all possible pairwise comparisons of strains was used for principal component analysis. As the first two principal components explain the majority of the variability in the strains, we used the first two principal components to exclude effects of population structure.

For each transcript, evolutionary rate was adjusted for the first two principal components and residuals were used as an estimate of population structure-adjusted evolutionary rate. For each pair of transcripts, correlation between the population structure-adjusted mutation rate across strains was computed as a measure of coevolution of transcripts.

To estimate the significance of the correlation, false discovery rate (FDR) was calculated using the R package FDRtool (Strimmer, 2008). FDRtool estimates FDR using a mixture model on the correlation coefficient with empirical parametric null-distribution and a nonparametric alternate distribution. Transcript pairs with FDR less than 0.1% were then selected to construct a network where nodes represent transcripts and edges represent significant evidence for coevolution. Clusters were identified from a network by extracting components, defined as a subset of transcripts where any pair of transcripts is connected by at least one path in the network.

In addition, we used linear mixed model as implemented in R package EMMA (Kang et al., 2008) to correct for the population structure. We used EMMA to calculate association between all possible pairwise combinations of transcript using evolutionary rate matrix as generated above. The transcript pairs showing significant association between them were selected and networks were constructed as described above.

Network Overlap

The overlap between PCA-based and EMMA-based networks was computed by taking the ratio of the number of common edges to the minimum of the number of edges in both networks. Overlap was evaluated by considering that a fixed number of edges was independently sampled in each network and significance evaluated using Fisher's exact test. When considering clusters unique to a single strain, the overlap was computed as the fraction of edges from the PCA network compared to those present in the EMMA network. All the analysis was performed in R.

Overlap with Genome-wide Association Studies

We screened all genome-wide association (GWA) studies recorded by the National Human Genome Research Institute (www.genome.gov/gwastudies) on or before 26 October 2012 for relevant phenotypes (Table S8). Of all annotated rat transcripts, 27,199 had at least one one-to-one or one-to-many orthologs in the human genome, of which 1,499 had been shown to be associated with hypertension or metabolism-related phenotypes at a p value threshold of $p < 10^{-5}$.

Identification of Putative Artificial Selective Sweep Regions

To identify genomic regions shared between different rat strains, relative SNV density (RSD) in nonoverlapping 10kb windows was calculated. For pairs of genomes from substrains that arose from the same parental strains (SS/JrHsdMcwi and SS/Jr; LEW/NCrlBR and LEW/Crl; SHR/NHsd and SHR/OlaIpcv), SNVs from pair of substrains were pooled. For WKY-derived strains however, WKY/Gla showed substantial diversity from the other two WKY substrains and we therefore considered WKY/Gla as a separate strain and only pooled SNVs from the other two WKY strains. This defined a set of 22 genomes for selective sweep analysis. We excluded all polymorphic loci with missing genotypes and/or heterozygous calls in at least one strain, defining a set of 7.8 million high quality SNVs (out of a total of 9.6 million SNVs) that were used for further analysis.

For each 10 kb window in each strain relative SNV density (RSD) was calculated using the following formula:

$$\mathrm{RSD}i = \frac{\text{Number of SNVs in strain } i}{\text{Total number of SNVs}}\ x\ 100$$

In cases where only one strain shows a set of SNVs that are different from the reference strain, but all other strains show the reference allele at every site across the 10 kb window, the RSD value for that strain would be 100 and zero for other strains. If two strains show alternate (identical or nonidentical) SNV sets with equal numbers of SNVs in each strain and other strains show reference alleles, the expected RSD value would be 50 for those two strains and zero for other strains; for three strains showing equal numbers of alternate SNVs, the RSD would be 33.33 and so on. Chi-square statistics were used to determine the goodness of fit to a model of equal distribution of SNVs within each 10 kb window between 'n' strains. Chi-square statistics were calculated for all possible combinations of strains according to:

$$SC = \min\left(\binom{n}{k = 1\ldots n}\sum_{i=1}^{k}\frac{(O_i - E_i)^2}{E_i}\right)$$

The combination of RSDs which showed the lowest chi-square value for each 10 kb window was selected as the best fit model of RSD distribution across the 22 genomes.

Segments were then merged if successive 10 kb windows were shared between same strain combinations. Further smoothing was applied if two adjacent windows on both sides were shared between the same strain combinations.

We also estimated the probability of occurrence of long genomic regions that were unique to a given strain or to a number of strain combinations using a permutation test (n=10 million). Strain combinations assigned to each 10 kb window was shuffled by keeping the composition intact. For each iteration, we estimated the maximum length of segment assigned to the same strain or strain combination. FDR was calculated to correct for multiple testing using FDRtools (Strimmer, 2008) and genomic segments with FDR less than 0.1% were selected.

Calculation of Linkage Distribution

Linkage distribution between the SNVs within the coevolutionary gene clusters and SNVs putative selective sweeps was calculated using Haploview 4 (Barrett et al., 2005).

Coalescent Simulations

To assess the significance of identified PASS regions and to validate the statistical method used to show evidence for selection, we performed coalescent simulations using the software package msms (Ewing and Hermisson, 2010). To assess the significance of selective sweep signals, we generated a null model without selection but taking into account the known demographic history of rat strains sequenced (Ben-Ishay et al., 1972; Bianchi et al., 1974; Dupont et al., 1973; Goto et al., 1975; Jackson et al., 1981; Jong, 1984; Kuijpers and Gruys, 1984; Lindsey, 1979; Okamoto and Aoki, 1963; Okamoto et al., 1974). Demographic history was available for all the rat strains, including the year of derivation. The size of the originating colony and the number of starting breeding pairs were available for every disease model and their respective controls except for FHH and FHL where the colony size was

estimated at 500 and the number of breeding pairs estimated at 10. For the five remaining strains, breeding pair number was known for two and originating colony size was unknown. In these cases, breeding pair numbers were estimated at 10 and colony size at 500. Unknown branch points in the demographic history were estimated from the phylogenetic tree.

Rat strains were derived from multiple founder colonies and, moreover, rat strains derived from the same outbred founder colony were derived at different geographical locations where subsets of the founder colonies were distributed and then maintained. Each rat strain was derived either from a single pair of rats or a relatively small number of pairs and therefore underwent severe bottleneck effects between 100 and 200 generations ago. We considered each rat strain as a distinct population that went through a bottleneck and simulated the likelihood of unique or shared haplotype blocks for the 22 rat strains conditioned on the time point of selection and size of selected colony as reported. For the simulations we considered an average recombination rate 0.60 cM/Mb (Jensen-Seaman et al., 2004) and mutation rate of 2 x10^{-8} per base per generation and estimated an average of three generations per year. We generated 10,000 chromosomes each with 30,000 polymorphic sites corresponding to a genomic segment of length 10Mb.

The following commands were used for generating null model:

java –Xmx20G –jar msms3.2rc-b80.jar -N 10000–ms 22 10000 -t 0.0008 -r 720 -s 30000 -I 22 1 1 1 1 1 1 1 1 1 1 1 1 1 1 1 1 1 1 1 1 1 1 -en 0.0025 19 0.00025 -en 0.0025 20 0.00025 -ej 0.002525 20 19 -en 0.002525 19 0.0125 -en 0.003125 13 0.00005 -en 0.003125 14 0.00005 -en 0.003125 15 0.00005 -en 0.003125 17 0.00125 -en 0.003125 18 0.00125 -ej 0.00315 13 14 -ej 0.00315 17 18 -en 0.00315 18 0.0125 -ej 0.003175 14 15 -en 0.003175 15 0.00375 -en 0.00325 8 0.0025 -en 0.00325 5 0.005 -en 0.00325 1 0.005 -en 0.003275 5 0.005275 -en 0.003275 1 0.005275 -en 0.00325 8 0.025 -en 0.003375 6 0.00025 -en 0.003375 7 0.00025 -en 0.003375 3 0.0025 -ej 0.0034 6 7 -en 0.0034 7 0.0125 -en 0.0034 4 0.0025 -ej 0.0034 3 2 -en 0.0034 2 0.005275 -en 0.00375 2 0.00005 -en 0.00375 11 0.0025 -en 0.00375 12 0.0025 -ej 0.00375 11 12 -en 0.003775 12 0.025 -en 0.003775 2 0.0017 -en 0.004375 10 0.0025 -en 0.0044 10 0.025 -ej 0.0045 15 12 -ej 0.005 4 2 -en 0.005 2 0.5 -ej 0.005625 2 1 -en 0.005625 1 1 -ej 0.00625 5 1 -ej 0.0068 10 9 -ej 0.0069 9 8 -ej 0.007 8 7 -ej 0.007125 7 12 -ej 0.0075 12 1 -en 0.0075 1 2.25 -en 0.01 16 0.00025 -en 0.010025 16 0.025 -ej 0.010125 16 1 -ej 0.010625 18 1 -ej 0.0107 19 1 -ej 0.0125 21 1 -ej 0.0125 22 1 -en 0.0125 1 4.5 -threads 8

To validate further the statistical method used to identify putative selective sweeps, we generated two models, one with selection and one corresponding to the null model without selection. For both these models we generated 22 samples from a single population with constant population size. For each model we generated 105 chromosomes with 3000 polymorphic sites.

The following commands were used to generate these models

To Generate the Model in which Selection Occurred at a Single Locus

java –Xmx20G –jar msms3.2rc-b80.jar -N 10000 -ms 22 100000 -t 0.0008 -r 72 -s 3000 -SAA 2000 -SaA 1 -SF 0.01

To Generate the Corresponding Null Model for the Selection Model Above

java –Xmx20G –jar msms3.2rc-b80.jar -ms 22 100000 -t 0.0008 -r 72 -s 3000

In the sample of 10^5 simulated chromosomes, in 79.3% of the chromosomes selective sweep signals were also identified by the RSD analysis. All putative selective sweeps showed negative Tajima's D; 93.68% of the sweeps showed Tajima's D < -1.99 (FDR < 0.05).

Calculation of the Population Genetics Statistic Tajima's D

The population genetics statistic Tajima's D was calculated in 10kb nonoverlapping windows with a custom Perl script using the formula described in original paper (Tajima, 1989). False discovery rate (FDR) was calculated using the null distribution of Tajima's D obtained from the coalescent simulated null model:

$$FDR = P(H=0|D<d)$$

$$= \frac{P(D<d|H=0)\ x\ P(H=0)}{P(D<d)}$$

Where,

$$P(D<d|H=0) = \frac{no.\ of\ permutations < d}{no.\ of\ permutations}$$

$$P(D<d) = \frac{number\ observed < d}{number\ observed}$$

$$P(H=0) \leq 1$$

Overlap of Putative Artificial Selective Sweeps with Physiological Quantitative Trait Loci

We downloaded all physiological quantitative trait loci (pQTLs) reported in RGD (http://rgd.mcw.edu/) and extracted the pQTLs for hypertension and metabolic phenotypes. We used pQTL intervals as defined in RGD. We define overlap between pQTL and artificial selective sweeps if the following criteria were satisfied. (1) The start and end of the selective sweep must be within the pQTL interval

as defined by RGD. (2) The pQTL must be mapped in the same strain in which the putative selective sweep was identified. Overlap between pQTLs and PASS regions was estimated conditioned on above criteria using a Perl script and the significance was estimated by permutation testing.

Gene Ontology Analysis

Gene ontology enrichment and KEGG pathway enrichment analysis was carried out using The Database for Annotation, Visualization and Integrated Discovery (DAVID) v6.7 (Huang et al., 2009).

Data Availability and Accession Numbers

All sequence reads from this study are deposited in the EBI Sequence Read Archive under accession number ERP002160 (http://www.ebi.ac.uk/ena/data/view/ERP002160). All sequence variants are deposited in RGD (http://rgd.mcw.edu/). LE/Stm BAC sequences are deposited in EBI-EMBL under accession number FO117624- FO117632 and FO181540- FO181543.

SUPPLEMENTAL REFERENCES

Ben-Ishay, D., Saliternik, R., and Welner, A. (1972). Separation of two strains of rats with inbred dissimilar sensitivity to Doca-salt hypertension. Experientia *28*, 1321–1322.

Bianchi, G., Fox, U., and Imbasciati, E. (1974). The development of a new strain of spontaneously hypertensive rats. Life Sci. *14*, 339–347.

Dupont, J., Dupont, J.C., Froment, A., Milon, H., and Vincent, M. (1973). Selection of three strains of rats with spontaneously different levels of blood pressure. Biomedicine *19*, 36–41.

Goto, Y., Kakisaki, M., and Masaki, N. (1975). Spontaneous diabetes produced by repeated selective breeding of normal Wistar rats. Proc. Jpn. Acad. *51*, 80–85.

Huang, W., Sherman, B.T., and Lempicki, R.A. (2009). Systematic and integrative analysis of large gene lists using DAVID bioinformatics resources. Nat. Protoc. *4*, 44–57.

Jackson, R., Rassi, N., Crump, T., Haynes, B., and Eisenbarth, G.S. (1981). The BB diabetic rat. Profound T-cell lymphocytopenia. Diabetes *30*, 887–889.

Jensen-Seaman, M.I., Furey, T.S., Payseur, B.A., Lu, Y., Roskin, K.M., Chen, C.F., Thomas, M.A., Haussler, D., and Jacob, H.J. (2004). Comparative recombination rates in the rat, mouse, and human genomes. Genome Res. *14*, 528–538.

Kuijpers, M.H., and Gruys, E. (1984). Spontaneous hypertension and hypertensive renal disease in the fawn-hooded rat. Br. J. Exp. Pathol. *65*, 181–190.

Okamoto, K., and Aoki, K. (1963). Development of a strain of spontaneously hypertensive rats. Jpn. Circ. J. *27*, 282–293.

Okamoto, K., Yamori, Y., and Nagaoka, A. (1974). Establishment of the stroke-prone spontaneously hypertensive rat (SHR). Circ. Res. *34*(Suppl.), I-143–I-153.

Sherry, S.T., Ward, M.H., Kholodov, M., Baker, J., Phan, L., Smigielski, E.M., and Sirotkin, K. (2001). dbSNP: the NCBI database of genetic variation. Nucleic Acids Res. *29*, 308–311.

Strimmer, K. (2008). fdrtool: a versatile R package for estimating local and tail area-based false discovery rates. Bioinformatics *24*, 1461–1462.

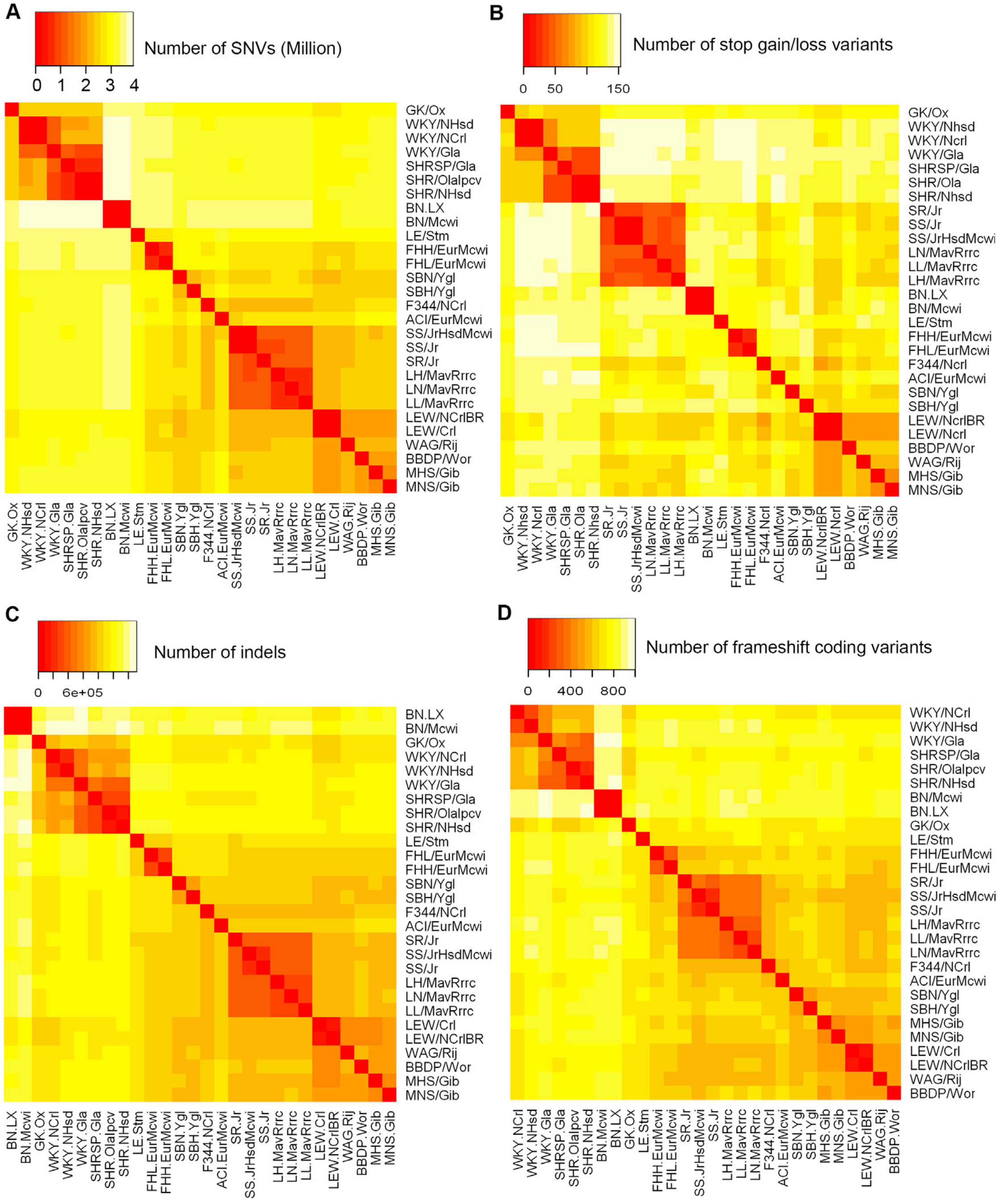

Figure S1. Pairwise Comparison of SNVs, Stop Gain or Stop Loss Variants, Indels, and Frameshift Coding Variants, Related to Table 1
Heat maps showing (A) Number of SNVs between any pair of 28 rat strains including the BN reference genome, calculated by counting the number of alleles differing between any pair of strains; (B) Number of stop gain or stop loss variants between any pair of 28 rat strains. (C) Number of Indels between any pair of 28 rat strains including the BN reference genome, calculated by counting the number of alleles differing between any pair of strains; (D) Number of frameshift coding variants between any pair of 28 rat strains.

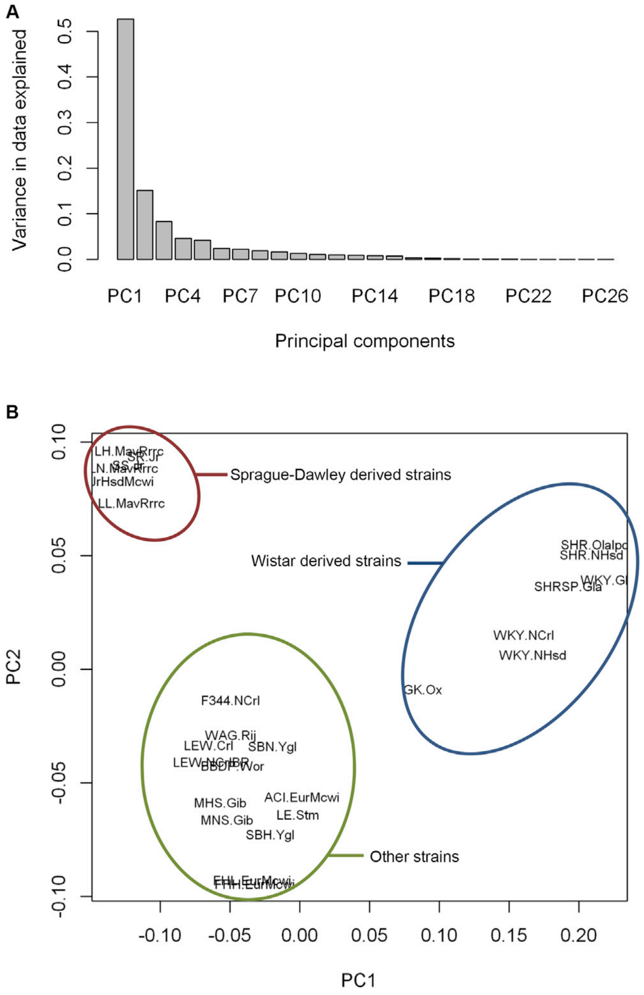

Figure S2. Principal Component Analysis of Rat Strains Sequenced, Related to Figure 2

(A) Principal component analysis shows that the first two principal components contribute to 70% of the sequence variability across the rat strains.

(B) The first two principal components separate Japanese Wistar-derived strains, Sprague-Dawley-derived strains and remaining rat strains.

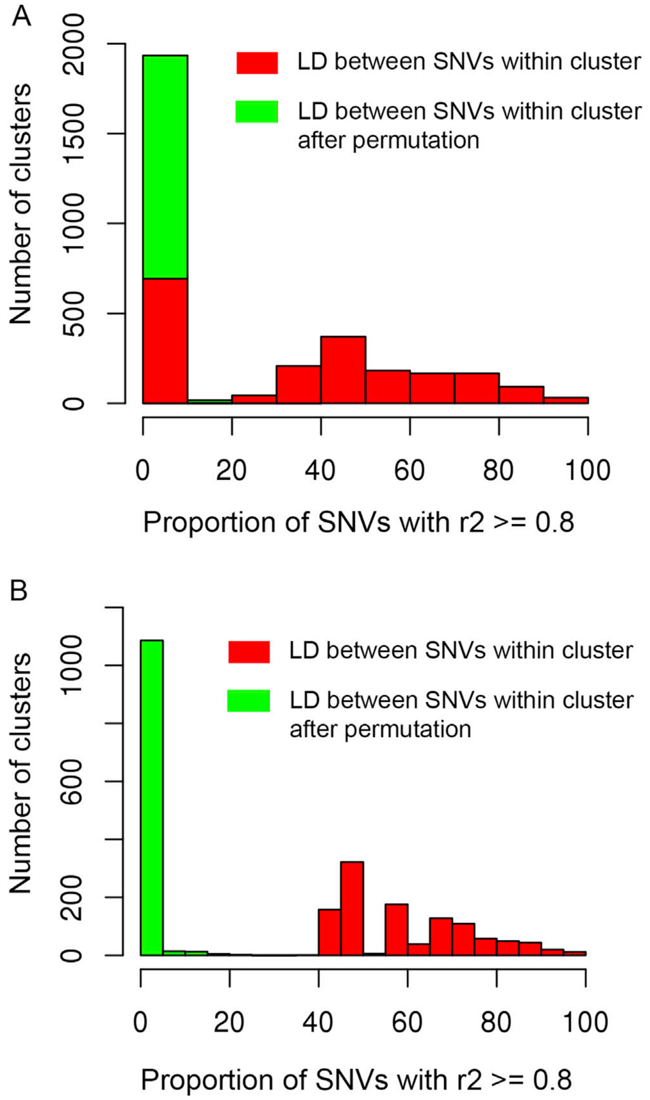

Figure S3. Linkage Disequilibrium in Coevolutionary Gene Clusters, Related to Table 2
Red bars show linkage disequilibrium (LD) between the SNVs in the genes within the cluster while the green bar shows LD between the SNVs after random shuffling. A) LD structure in all the clusters; B) LD structure in the clusters where at least 40% of the SNVs in the cluster show strong LD (r2 > = 0.8).

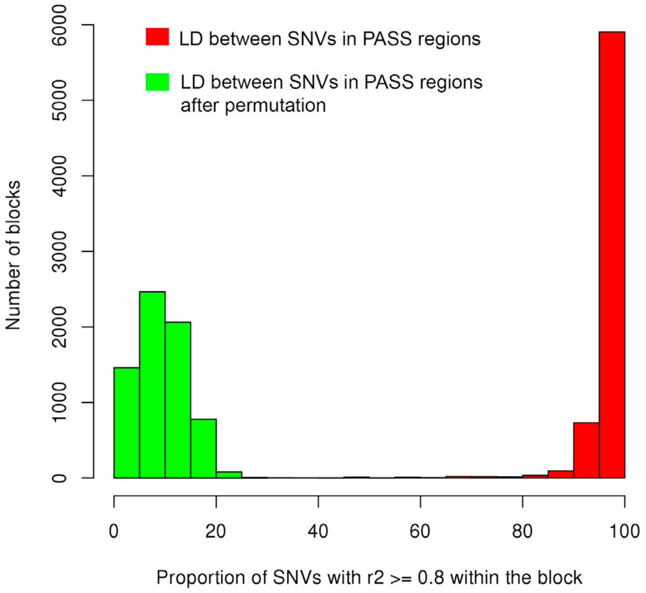

Figure S4. Linkage Disequilibrium in Putative Artificial Selective Sweep Regions, Related to Figure 3
Red bars show LD between the SNVs within the putative artificial selective sweeps: in the majority of the putative artificial selective sweep (PASS) regions almost all SNVs show strong LD. After random shuffling of all SNVs in the genomes of the 27 rat strains, the strong LD between SNVs in PASS regions is completely lost (represented as green bars).
